# Coupling of Peptidoglycan Synthesis to Central Metabolism in Mycobacteria: Post-transcriptional Control of CwlM by Aconitase

**DOI:** 10.1016/j.celrep.2020.108209

**Published:** 2020-09-29

**Authors:** Peter J. Bancroft, Obolbek Turapov, Heena Jagatia, Kristine B. Arnvig, Galina V. Mukamolova, Jeffrey Green

**Affiliations:** 1 Molecular Biology and Biotechnology, University of Sheffield, Sheffield, S10 2TN, UK; 2 Leicester Tuberculosis Research Group, Department of Respiratory Sciences, University of Leicester, Maurice Shock Medical Sciences Building, University Road, Leicester, LE1 9HN, UK; 3 Institute for Structural and Molecular Biology, University College London, London, WC1E 6BT, UK

**Keywords:** aconitase, CwlM, iron-responsive element, *Mycobacterium tuberculosis*, nitrosative stress, oxidative stress, peptidoglycan, post-transcriptional regulation, protein kinase B (PknB), macrophage infection

## Abstract

*Mycobacterium tuberculosis* causes human tuberculosis, and a better understanding of its biology is required to identify vulnerabilities that might be exploited in developing new therapeutics. The iron-sulfur cluster of the essential *M. tuberculosis* central metabolic enzyme, aconitase (AcnA), disassembles when exposed to oxidative/nitrosative stress or iron chelators. The catalytically inactive apo-AcnA interacts with a sequence resembling an iron-responsive element (IRE) located within the transcript of another essential protein, CwlM, a regulator of peptidoglycan synthesis. A *Mycobacterium smegmatis cwlM* conditional mutant complemented with *M. tuberculosis cwlM* with a disrupted IRE is unable to recover from combinations of oxidative, nitrosative, and iron starvation stresses. An equivalent *M. tuberculosis cwlM* conditional mutant complemented with the *cwlM* gene lacking a functional IRE exhibits a growth defect in THP-1 macrophages. It appears that AcnA acts to couple peptidoglycan synthesis and central metabolism, and disruption of this coupling potentially leaves mycobacteria vulnerable to attack by macrophages.

## Highlights

• Mutation of the *M. tuberculosis cwlM* IRE impairs binding by apo-aconitase
• Apo-aconitase:*cwlM* IRE interactions couple metabolism and peptidoglycan synthesis
• Decoupling by mutation of the *cwlM* IRE sensitizes *M. smegmatis* to host-related stresses
• Mutation of the *cwlM* IRE in *M. tuberculosis* results in a growth defect in macrophages

## Graphical Abstract

**Figure:**
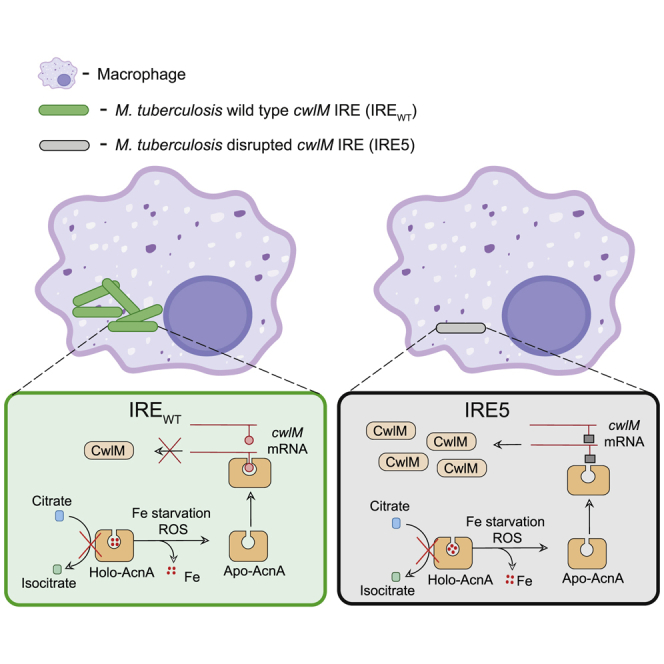

## Teaser

Bancroft et al. show that host-associated stresses cause disassembly of the iron-sulfur cluster of *Mycobacterium tuberculosis* aconitase. The resulting apo-aconitase binds an IRE-like sequence within the *cwlM* transcript. Disruption of this interaction enhances the sensitivity of mycobacteria to iron starvation and nitrosative and oxidative stresses and impairs growth in macrophages.

## Introduction

Aconitase (Acn) is an iron-sulfur protein that catalyzes the inter-conversion of citrate and isocitrate via *cis*-aconitate in the Krebs and glyoxylate cycles (Artymiuk and Green, 2006). Three cysteine residues coordinate three Fe ions of a [4Fe-4S] cluster located at the Acn active site. The uncoordinated Fe ion of the cluster (Fe_a_) binds the substrate and acts as a Lewis acid in the *trans*-elimination of H_2_O from citrate or isocitrate, yielding *cis*-aconitate (Emptage et al., 1983). Depending on the manner in which *cis*-aconitate then binds to Fe_a_ (either in citrate mode or isocitrate mode), rehydration produces either citrate or isocitrate (Lauble et al., 1994). Remarkably, some Acn proteins are bi-functional, acquiring the ability to bind specific mRNA molecules when the [4Fe-4S] cluster is lost (Goss and Theil, 2011). These apo-Acns are formed during prolonged Fe starvation or exposure to reactive oxygen species (ROS) or reactive nitrogen species (RNS) (Tong and Rouault, 2007; Soum and Drapier, 2003). The best characterized bi-functional Acns are the eukaryotic cytoplasmic Acns, also known as IRP1 (iron-responsive protein 1) (Artymiuk and Green, 2006). IRP1s bind to stem-loop structures known as iron-responsive elements (IREs) that are located in the untranslated regions (UTR) of mRNA molecules (Walden et al., 2006, 2012). Generally, binding of IRP1s at IREs located within the 3′ UTR stabilizes the mRNA and promotes translation, whereas binding at the 5′ UTR inhibits translation (Goss and Theil, 2011). Bacterial AcnA proteins are closely related to the eukaryotic IRP1s and are also bi-functional (Gruer et al., 1997). AcnA proteins have been reported to regulate their own expression and sporulation, co-ordinate the oxidative stress response, and control virulence gene expression in Gram-positive and Gram-negative bacteria (Wilson et al., 1998; Alén and Sonenshein, 1999; Tang and Guest, 1999; Serio et al., 2006; Michta et al., 2014; Austin et al., 2015). The *acn* gene of the major global pathogen *Mycobacterium tuberculosis* is essential (DeJesus et al., 2017) and codes for a bi-functional AcnA. The apo form of *M. tuberculosis* AcnA has been reported to interact with IRE-like sequences in the *trxC* (a thioredoxin) and *ideR* (iron-dependent transcriptional repressor) mRNAs (Banerjee et al., 2007).

*M. tuberculosis* causes human tuberculosis (TB), which is the leading cause of death from a single infection. It is estimated that 1.3 million deaths were caused by TB as a single infection in 2018, and a further 0.3 million deaths occurred among those with human immunodeficiency virus (HIV) infection (World Health Organization, 2018). Although a healthy immune system can contain *M. tuberculosis* in a pulmonary granuloma, it cannot efficiently kill bacteria that have transitioned into a non-replicating (dormant) state. Up to 1.7 billion people carry latent TB infections, of whom 5%–10% will develop active TB during their lifetimes, usually as a consequence of a compromised immune system (Guirado et al., 2013; World Health Organization, 2018). *In vitro* studies have shown that dormancy can be induced when mycobacteria are stressed by oxygen starvation, iron starvation, ROS, or RNS, all of which are experienced within the granuloma (Wayne and Sohaskey, 2001; Betts et al., 2002; Voskuil et al., 2003; Kurthkoti et al., 2017). Mycobacterial dormancy is also characterized by alterations to peptidoglycan synthesis and the structure of the cell wall (Dulberger et al., 2020). Although the precise role of cell wall remodeling is unclear, it is believed to be required for transition to, and resuscitation from, dormancy. Resuscitation from dormancy is defined as the restoration of typical mycobacterial growth and metabolism. *M. tuberculosis* possesses five genes (*rpfA*–*rpfE*) coding for resuscitation-promoting factors (Rpfs), which are secreted growth factors that are able to increase the number of viable cells recoverable from dormant non-replicating cultures (Mukamolova et al., 2002). Rpfs are transglycosylases, and hence it was suggested that they act by remodeling peptidoglycan structure (Cohen-Gonsaud et al., 2005). Rpfs are thought to be required for virulence, suggesting that remodeling of *M. tuberculosis* peptidoglycan is important during infection (Kana et al., 2008; Rosser et al., 2017), and their expression is tightly regulated both transcriptionally and post-transcriptionally (Schwenk and Arnvig, 2018). Among the complex repertoire of proteins involved in regulating peptidoglycan synthesis in *M. tuberculosis* is CwlM, an essential cytoplasmic protein similar to peptidoglycan amidases (Boutte et al., 2016; Turapov et al., 2018). At least two forms of CwlM are present in mycobacteria. The native, non-phosphorylated form is membrane associated and makes a complex with the proposed lipid II flippase MurJ, presumably to regulate the translocation of peptidoglycan precursors across the cytoplasmic membrane (Turapov et al., 2018). Phosphorylation of CwlM (to yield CwlM∼P) by the serine-threonine protein kinase PknB results in CwlM∼P-mediated activation of MurA, the first enzyme in peptidoglycan precursor synthesis (Boutte et al., 2016; Dulberger et al., 2020) and the formation of a cytosolic complex with the forkhead-associated domain protein FhaA (Turapov et al., 2018). MurJ is also a PknB substrate and upon phosphorylation also forms a complex with FhaA (Gee et al., 2012). Therefore, CwlM is thought to regulate *M. tuberculosis* peptidoglycan precursor synthesis and subsequent translocation across the cytoplasmic membrane during growth. However, the mechanisms for the rapid shut-down of peptidoglycan synthesis that allows *M. tuberculosis* to survive in the hostile environment generated by the host immune system are currently unknown.

Here, we show that the IRE that was thought to reside in the 3′ UTR of the *trxC* transcript (Banerjee et al., 2007) is in fact located within the 5′ region of the *cwlM* open reading frame. Recombinant *M. tuberculosis* AcnA was isolated with a [4Fe-4S] cluster and behaved as a typical Acn enzyme. Acn activity was lowered by disassembly of the iron-sulfur cluster upon exposure to ROS and/or the iron chelator bipyridyl (BIP) but was more resistant to RNS. The apo form of AcnA was able to bind specifically to the IRE within the *cwlM* transcript. Site-directed mutagenesis to disrupt the *cwlM* IRE without changing the amino acid sequence of the CwlM protein impaired apo-AcnA binding. A conditional *Mycobacterium smegmatis cwlM* mutant was complemented with either wild-type (WT) *M. tuberculosis cwlM* (*cwlM*_*Mt*_) or *cwlM* lacking a functional IRE (*cwlM*_*Mt*_-IRE5). The latter strain, henceforth referred to as *Ms*-IRE5, exhibited increased sensitivity to combinations of ROS, RNS and iron starvation compared with a strain complemented with the WT *cwlM*_*Mt*_ gene (*Ms*-IRE_WT_). This phenotype was associated with a failure to downregulate the intracellular level of CwlM protein, leading to sustained accumulation of non-phosphorylated CwlM. Similarly, a conditional *M. tuberculosis cwlM* mutant complemented with *cwlM*_*Mt*_ containing a non-functional IRE (*Mt*-IRE5) exhibited impaired growth compared with the strain complemented with the native *cwlM*_*Mt*_ (*Mt*-IRE_WT_) in THP-1 macrophages. Our results suggest that the iron-sulfur cluster of *M. tuberculosis* AcnA is a sensor of host-imposed oxidative stress and iron starvation, and under such conditions the disassembly of the [4Fe-4S] cluster switches AcnA from an enzyme of the Krebs cycle to a post-transcriptional regulator of *cwlM* expression. The interaction between apo-AcnA and the *cwlM* IRE reveals a critical function of a bacterial IRE in permitting intracellular growth. Moreover, it provides a mechanism to couple central metabolism to peptidoglycan synthesis and exposes a potential vulnerability in *M. tuberculosis* defenses if, in the future, small-molecule disrupters of the *cwlM*, and other IREs, could be developed.

## Results

### Apo-AcnA Recognizes an IRE-like Sequence within the Coding Region of the *M. tuberculosis cwlM* Transcript

**Figure 1:**
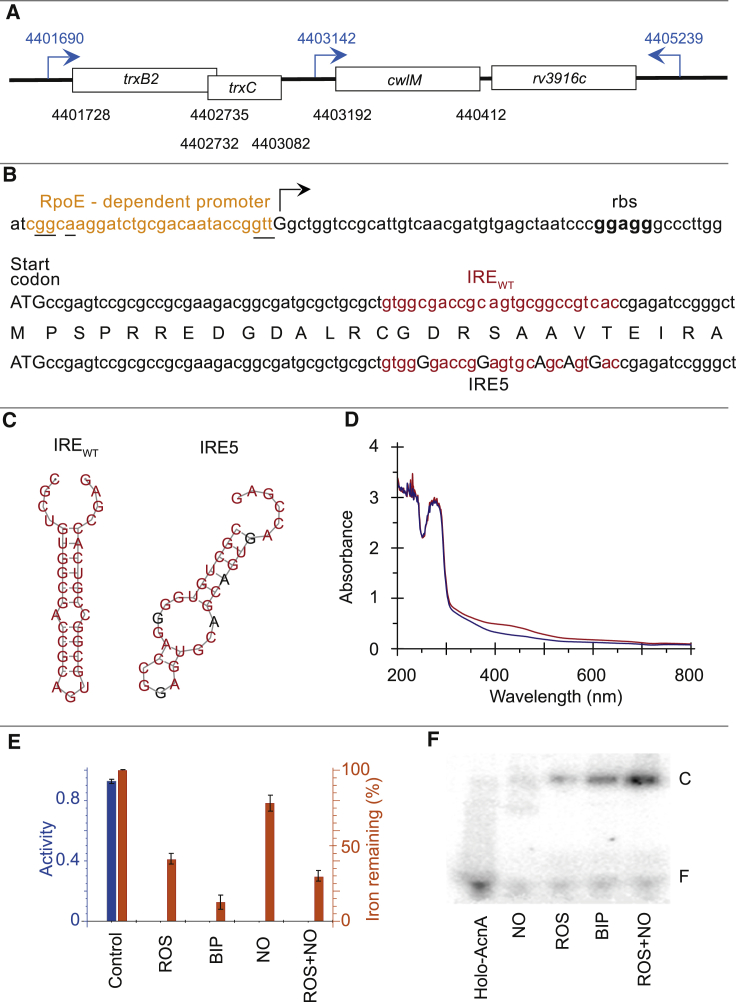
The *M. tuberculosis cwlM* Transcript Possesses an IRE that Is Recognized by Apo-AcnA (A) Map of the *cwlM* genetic locus of *M. tuberculosis* H37Rv showing open reading frames (rectangles) and their genome coordinates (black type) and primary transcript start points (blue arrows) and their coordinates (blue type). The *trxB2-trxC* genes are arranged as an operon with the *trxC* start codon located within the *trxB2* open reading frame. (B) DNA sequence of *cwlM* regulatory elements. A potential RpoE-dependent promoter with bases matching the consensus sequence underlined (brown type; consensus: gGGAACYa-[N_15-16_]-cGTT) (Manganelli, 2014); the experimentally determined *cwlM* transcript start is arrowed (uppercase) (Cortes et al., 2013); the ribosome binding site (rbs; bold black type) and start codon (ATG) are indicated; the native iron-responsive element (IRE_WT_; red type) and a mutated IRE (IRE5) that has five base replacements (uppercase, black type) that disrupt the IRE structure but maintain the amino acid sequence of CwlM (single-letter code; uppercase) are shown. (C) Predicted structures of the *cwlM* native IRE (IRE_WT_, left) and the mutated IRE5 (right) generated by RNA fold (Gruber et al., 2008). The native bases are shown in red; mutated bases are shown in black. (D) UV-visible spectra of holo-AcnA (red trace) and apo-AcnA (blue trace). Broad absorbance around 420 nm is indicative of the presence of an iron-sulfur cluster. (E) AcnA activity (nanomoles NADPH produced min^−1^ μg^−1^ AcnA; blue bars) and iron content (orange bars) are decreased by exposure to superoxide (ROS), the iron chelator bipyridyl (BIP), nitric oxide (NO), or a combination of superoxide and nitric oxide (ROS+NO). Data are represented as mean ± SD (n = 3). (F) Exposure of holo-AcnA to nitric oxide (NO), superoxide (ROS), bipyridyl (BIP), or a combination of superoxide and nitric oxide (ROS+NO) promotes binding of AcnA to the *cwlM* IRE sequence. Electrophoretic mobility shift assays were carried out as described in STAR Methods. The locations of the free, unbound labeled IRE (F) and the AcnA-IRE complex (C) are indicated. Complexes were detected after treating AcnA with the nitric oxide donor spermine NONOate (NO), xanthine oxidase-generated superoxide (ROS), bipyridyl (BIP), or the combination of superoxide and NO (ROS+NO). See also Figure S1.

Previous work identified a potential IRE in the *trxB2-trxC-cwlM-Rv3916c* locus of *M. tuberculosis* H37Rv (Banerjee et al., 2007). The IRE was thought to be located in the 3′ UTR of the *trxC* transcript, and binding of apo-AcnA to the IRE was demonstrated (Banerjee et al., 2007). However, analysis of the *M. tuberculosis* H37Rv global transcription start site datasets reported by Cortes et al. (2013) revealed that the IRE was in fact located within the *cwlM* coding sequence (codons 14–22) (Figures 1A–1C). Promoter sequence analysis suggested that *cwlM* transcription might initiate from an RpoE-dependent promoter (Figure 1B; Manganelli, 2014).

**Figure 2:**
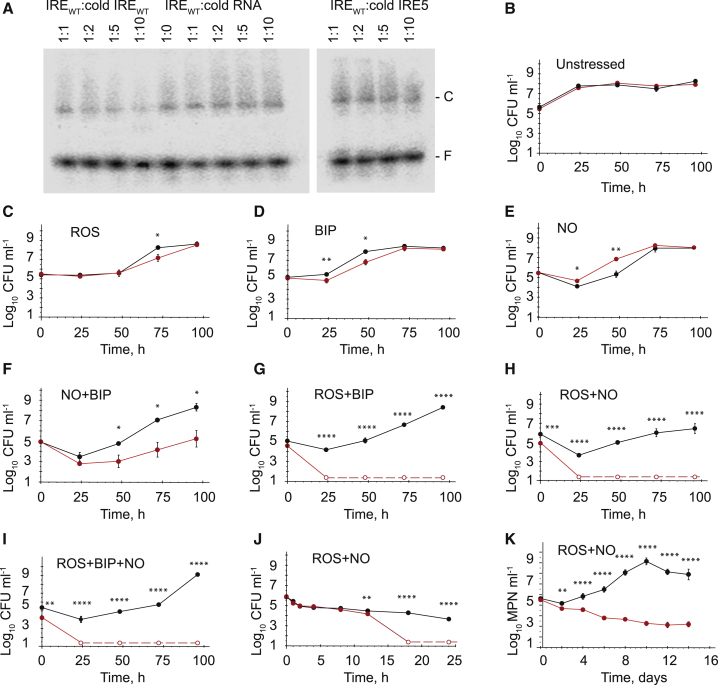
Growth of *Mycobacterium smegmatis* with a Disabled *cwlM* IRE Is Impaired When Challenged by Host-Associated Stresses (A) Apo-AcnA binds specifically to the *cwlM* IRE. Representative competition EMSAs are shown in which apo-AcnA (20 μM) was incubated with radiolabeled *cwlM* IRE_WT_ (10 ng) in the presence of either cold IRE_WT_ (IRE_WT_:cold IRE_WT_), cold yeast RNA (IRE_WT_:cold RNA), or cold *cwlM* IRE5 (IRE_WT_:cold IRE5) at the ratios indicated above each lane. The locations of the free radiolabeled IRE (F) and the AcnA-IRE complex (C) are indicated. (B–K) Survival of *M. smegmatis* exposed to single or combinations of oxidative stress, nitrosative stress, and iron starvation. Strains of *M. smegmatis cwlM*-CM were grown in 7H9 broth to OD_600_ ~ 0.8, then diluted 1:1,000 in 50 ml fresh broth containing the stress reagents. For the experiments shown in (B)–(K), triplicate cultures of *Ms cwlM*-CM complemented with either the native *cwlM*_*Mt*_ gene (black lines and circles) or *cwlM*_*Mt*_-IRE5, which has a disrupted IRE (red lines and circles), were incubated at 37°C under aerobic conditions. Growth of untreated cultures (B) or the cultures treated with (C) plumbagin (75 μM; ROS); (D) bipyridyl (100 μM; BIP); (E) spermine NONOate (200 μM; NO); (F) NO and BIP (NO+BIP); (G) ROS and BIP (ROS+BIP); (H) ROS and NO (ROS+NO); or (I) ROS, BIP, and NO (ROS+BIP+NO) was followed for 96 h by measuring CFU at 24, 48, 72, and 96 h on 7H10 agar. For the zero time point measurements, CFU counts were determined immediately after addition of the stress agents. Several combination stresses resulted in the recovery of zero CFU for the *Ms*-IRE5 strain after 24 h (G–I); therefore these points (shown as open circles joined by dashed lines) correspond to the limit of detection (24 CFU ml^−1^). (J) Response of bacterial cultures subjected to a combination of ROS and NO (ROS+NO) stresses for 24 h. For zero time point, CFU counts were measured before addition of chemicals. (K) Estimation of the population of “non-plateable” bacteria using most probable number (MPN) assays for over a 14-day period. In (B)–(K), data are represented as mean ± SEM (n = 3). Unpaired t test was used for assessment of significant difference of viable counts between *cwlM*_*Mt*_ and *cwlM*_*Mt*_-IRE5 expressing *M. smegmatis*. CFU counts of *Ms cwlM*-CM complemented with native *cwlM* (black) were significantly different from *cwlM*-IRE5 (red) counts (^∗^p < 0.05, ^∗∗^p < 0.01, ^∗∗∗^p < 0.001, and ^****^p < 0.0001, unpaired t test). See also Figure S2.

The *M. tuberculosis* H37Rv *acn* gene was cloned into plasmid pET28, and the protein, AcnA, was expressed in *Escherichia coli* BL21 (λDE3) and purified. Recombinant AcnA protein had an iron content (5.7 ± 0.6 iron atoms per AcnA; n = 7) and a UV-visible spectrum consistent with the presence of a [4Fe-4S] cluster (Figure 1D). The catalytic parameters of *M. tuberculosis* AcnA were typical of an Acn enzyme, with V_max_ of 1.6 ± 0.1 U mg^−1^ (where 1 U is equal to 1 μmol of citrate converted to isocitrate per min) and K_M_ of 3.3 ± 0.2 mM, similar to those reported previously for bacterial AcnA proteins (Figure S1A; Jordan et al., 1999; Baothman et al., 2013). Acn activity decreased when AcnA was exposed to superoxide (ROS), nitric oxide (NO), or BIP, and the inhibition was associated with disassembly of the iron-sulfur cluster, as indicated by the lower iron contents of the treated AcnA protein (Figure 1E). Disruption of the holo-AcnA iron-sulfur cluster by treatment with ROS, BIP, a combination of superoxide and NO (ROS+NO), or to a lesser extent by NO permitted binding of the resulting apo-AcnA protein to the *cwlM* IRE sequence in electrophoretic mobility shift assays (EMSAs) (Figures 1F, S1B, and S1C). Competition EMSAs showed that unlabeled *cwlM* IRE competed with labeled IRE, whereas non-specific yeast RNA did not (Figure 2A). Supershift assays confirmed that the retarded species observed in the EMSAs was an apo-AcnA-IRE complex (Figure S1D). Thus, it was concluded that *M. tuberculosis* AcnA has two mutually exclusive functions: holo-AcnA is an essential enzyme of the Krebs and glyoxylate cycles, but under conditions that disassemble the active site [4Fe-4S] cluster, catalytic activity is lost, and the apo-AcnA binds specifically to an RNA sequence with an IRE-like structure.

### Mutation of the *cwlM* IRE Impairs Binding by Apo-AcnA and Inhibits Growth of *M. smegmatis* Exposed to Host-Associated Stresses

Site-directed mutagenesis of the native *cwlM* IRE (IRE_WT_) was undertaken to disrupt its structure without altering the amino acid sequence of the CwlM protein (Figures 1B and 1C). The mutated IRE (IRE5) did not compete with radiolabeled IRE_WT_ in EMSAs, indicating that apo-AcnA did not effectively interact with IRE5 (Figure 2A).

To investigate the phenotypic effects of disrupting the *cwlM* apo-AcnA:IRE interaction, a *M. smegmatis* conditional mutant (*Ms cwlM*-CM) was constructed. The chromosomal copy of *cwlM* in *Ms cwlM*-CM is under the control of a pristinamycin-inducible promoter, such that the strain was able to grow in the presence of pristinamycin, but not in its absence (Figure S2; Turapov et al., 2018). Under standard laboratory culture conditions, in the absence of pristinamycin, similar growth dynamics, measured as “plateable” colony-forming units (CFU), were observed for *Ms cwlM*-CM complemented with either the *M. tuberculosis cwlM* gene (*Ms*-IRE_WT_) or with *cwlM*_*Mt*_ with a disrupted IRE (*Ms*-IRE5) (Figure 2B). However, when low-CFU cultures (inoculated to ∼10^5^ CFU ml^−1^) were exposed to plumbagin (oxidative stress, ROS) or BIP (iron restriction) the growth of *Ms*-IRE5 was impaired, with ∼10-fold fewer CFU ml^−1^ (p ≤ 0.05) being recovered at 48 h (BIP) and 72 h (ROS) after treatment compared with *Ms*-IRE_WT_ (Figures 2C and 2D). For cultures treated with the NO donor spermine NONOate (NO), the *cwlM*_*Mt*_-IRE5 complemented strain recovered more rapidly than *Ms*-IRE_WT_ (∼10-fold more CFU ml^−1^ were recovered 48 h after treatment of *Ms*-IRE5; p ≤ 0.01), suggesting that AcnA-mediated regulation of *cwlM* impaired recovery during nitrosative stress (Figure 2E). Overall, although the effects of applying individual stresses on the growth phenotype of the *Ms*-IRE5 strain were significant, they were modest. In contrast, exposure to combined stresses, consisting of spermine NONOate and BIP (NO+BIP; Figure 2F), plumbagin and BIP (ROS+BIP; Figure 2G), plumbagin and spermine NONOate (ROS+NO; Figure 2H), or plumbagin, BIP, and spermine NONOate (ROS+BIP+NO; Figure 2I), resulted in severe attenuation of the *Ms*-IRE5 strain. Whereas the stress combinations caused an initial 10- to 100-fold decrease in CFU for *Ms*-IRE_WT_, followed by resumption of growth (Figures 2F–2I, black lines), the *Ms*-IRE5 strain was very sensitive to the combined stresses, such that with the exception of the NO+BIP combination, the “plateable” bacteria were below the limit of detection (24 CFU ml^−1^) 24 h after treatment (Figures 2G–2I, red lines). Therefore, it was concluded that the *Ms*-IRE5 strain was hypersensitive to the combination stresses tested. To monitor this sensitivity soon after exposure to the ROS+NO combination, cultures were sampled 1, 2, 4, 8, 12, 18, and 24 h after treatment. During the first 2 h of exposure, the *Ms*-IRE_WT_ and *Ms*-IRE5 strains responded similarly, exhibiting ∼10-fold decrease in CFU (Figure 2J). However, the hypersensitivity of the *Ms*-IRE5 strain became evident after 18 h, when the CFU fell below the detection limit, whereas CFU were readily detected, albeit ∼100-fold lower than the pre-exposure levels, for the WT cultures (Figure 2J). Hence, it appeared that the absence of the *cwlM* IRE sequence did not affect the initial response to the ROS+NO combination but was crucial for the subsequent recovery phase.

Most probable number (MPN) assays were used to estimate the “non-plateable” or “differentially culturable” population of *Ms*-IRE5 after exposure to the ROS+NO combination. These showed that after 24 h, the “non-plateable” population had decreased, but was not zero, and that it continued to decline over the following 13 days, suggesting that the bacteria lacking the *cwlM* IRE were unable to fully recover from ROS+NO imposed damage (Figure 2K). In contrast, complementation with the unaltered *cwlM*_*Mt*_ gene permitted recovery, manifest as a progressive increase in bacterial numbers over the course of the experiments (Figure 2K).

**Figure 3:**
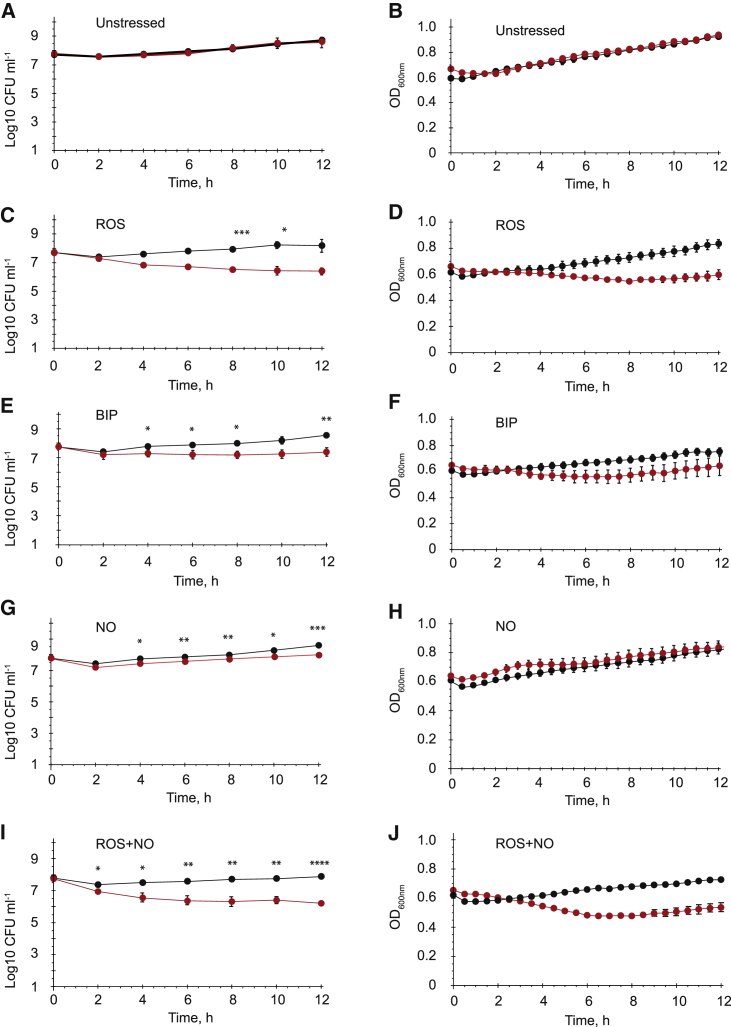
Disruption of the *cwlM* IRE Increases Sensitivity of *M. smegmatis* to a Combination of ROS and NO (A–J) Cultures of *Ms-cwlM*_WT_ and *Ms*-IRE5 were grown in 7H9 broth to OD_600_ ~ 0.6 before adding the stress reagents. Triplicate high-cell-density cultures (~5 × 10^7^ CFU ml^−1^) of *Ms cwlM*-CM complemented with native *cwlM* (black) or *cwlM*-IRE5 (red) were incubated under aerobic conditions at 37°C for 12 h. “Plateable” CFU and optical density at 600 nm were measured at regular intervals for (A and B) untreated; (C and D) ROS, treated with plumbagin (75 μM); (E and F) BIP, treated with bipyridyl (100 μM); (G and H) NO, treated with spermine NONOate (200 μM); and (I and J) ROS and NO, treated with plumbagin (75 μM) and spermine NONOate (200 μM). (A), (C), (E), (G), and (I) show CFU counts and data are presented as mean ± SEM (n = 3).; (B), (D), (F), (H), and (J) show optical density readings and data are presented as mean ± SD (n = 3). CFU counts of *Ms cwlM*-CM complemented with native *cwlM* (black) were significantly different from *cwlM*-IRE5 (red) counts (^∗^p < 0.05, ^∗∗^p < 0.01, ^∗∗∗^p < 0.001, and ^∗∗∗∗^p < 0.0001, unpaired t test).

Qualitatively similar results were obtained for cultures inoculated to a higher cell density (∼5 × 10^7^ CFU ml^−1^) before exposure to oxidative stress, iron starvation, or a combination of oxidative and nitrosative stresses: lower CFU were recovered for the *Ms*-IRE5 strain compared with the WT during the first 12 h of exposure to the stressors (Figure 3). Together these data indicated that the *cwlM* IRE is required for resisting combinations of oxidative, nitrosative, and iron starvation stresses in laboratory cultures.

### Disruption of the *cwlM* IRE Dysregulates CwlM Expression under Stress Conditions

**Figure 4:**
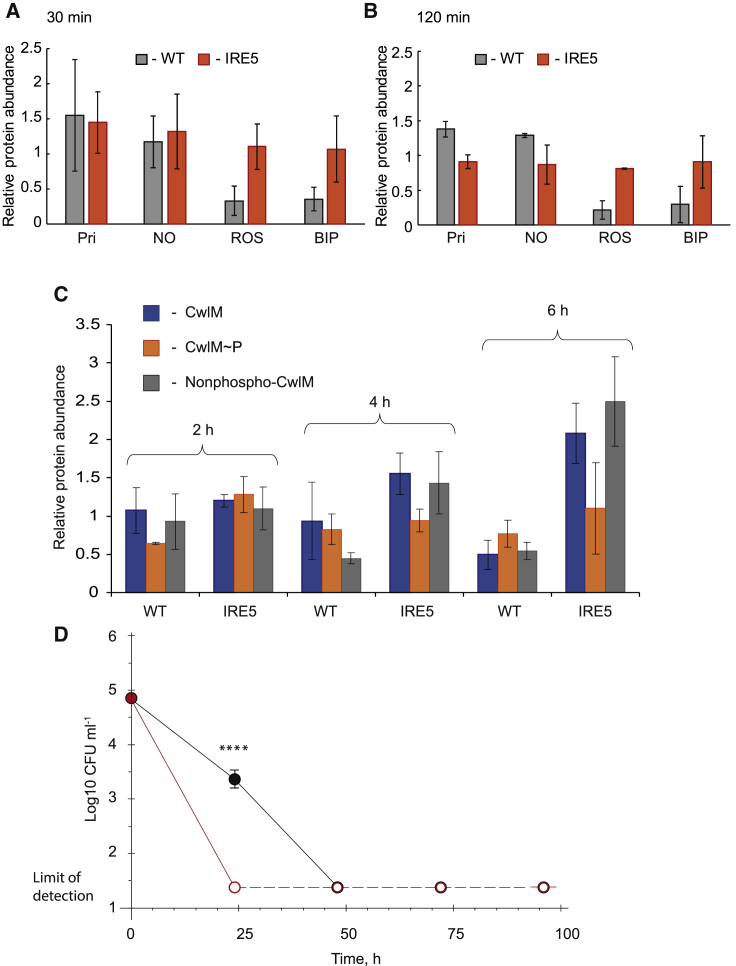
Disruption of the *cwlM* IRE Results in Increased Levels of Non-phosphorylated CwlM When Challenged with Combined Oxidative and Nitrosative Stresses (A and B) CwlM is downregulated in response to oxidative stress and iron starvation. Cultures of the *M. smegmatis cwlM* conditional mutant complemented with either unaltered *cwlM* (*Ms*-IRE_WT_; gray bars) or *cwlM* with a mutated IRE (*Ms*-IRE5; orange bars) were exposed to plumbagin (75 μM; ROS), spermine NONOate (200 μM; NO), or bipyridyl (100 μM; BIP), and samples were taken (A) 30 and (B) 120 min later. Western blots on cell lysates were developed with a polyclonal anti-CwlM antibody. In addition to the stressed cultures, untreated cultures were sampled as the normalizing control and cultures treated with pristinamycin (2 μg ml^−1^; Pri), to induce chromosomal *cwlM* expression, were analyzed. (C) Disruption of the *cwlM* IRE results in failure to downregulate CwlM 6 h after challenge with a combination of oxidative and nitrosative stresses. Cultures of *Ms*-IRE_WT_ or *Ms*-IRE5 were exposed to a combination of plumbagin (75 μM; ROS) and spermine NONOate (200 μM; NO). Samples were taken from two independent cultures at 2, 4, and 6 h after applying the stress. CwlM total protein was detected by polyclonal anti-CwlM antibody (blue bars), CwlM~P with anti-CwlM~P antibody (orange bars), and non-phosphorylated CwlM with anti-non-phosphorylated CwlM antibody (gray bars) (Turapov et al., 2018). In (A)–(C), data are represented as mean ± 95% confidence intervals (n = 2). Data obtained from both experiments are shown in Table S1. The raw images and the corresponding Coomassie blue-stained gels from the two independent cultures are provided in Figures S3–S5. (D) Triplicate cultures of *Ms*-IRE_WT_ (black lines and circles) or *Ms*-IRE5, which has a disrupted IRE (red lines and circles), were incubated at 37°C under aerobic conditions. The cultures were treated with pristinamycin (2 μg ml^−1^), to induce chromosomal *cwlM* expression, and plumbagin (75 μM; ROS) and spermine NONOate (200 μM; NO). Samples for CFU measurements were taken after 24, 48, 72, and 96 h; data are presented as mean ± SEM (n = 3). Zero CFU were recovered for the *Ms*-IRE5 strain after 24 h and for both strains after 48, 72, and 96 h; therefore these points (shown as open circles joined by dashed lines) correspond to the limit of detection (24 CFU ml^−1^). ^∗∗∗∗^ CFU counts of *Ms cwlM*-CM complemented with native *cwlM* (black) were significantly different from *cwlM*-IRE5 (red) counts. See also Tables 1 and S1 and Figures S3–S5.

Oxidative and nitrosative stresses are known to induce dramatic changes in *M. tuberculosis* gene expression (Nambi et al., 2015; Cortes et al., 2017). Therefore, expression of *cwlM* in *Ms*-IRE_WT_ and *Ms*-IRE5 was investigated after 1 h exposure to oxidative stress, nitrosative stress, iron starvation or a combination of nitrosative and oxidative stresses. Pristinamycin-induced cultures were included as positive controls. Samples were used for isolation of RNA for quantitative reverse transcription PCR (qRT-PCR) assays and preparation of lysates for western blot analysis. As expected, addition of pristinamycin increased *cwlM* expression and resulted in higher relative abundance of CwlM protein detected by western blotting using anti-CwlM antibody (Table 1). NO treatment or the ROS+NO combination resulted in a significant increase in the relative abundance of the *cwlM* transcript in both strains (p < 0.001), and western blot quantifications confirmed higher protein abundance (Table 1). Upon oxidative stress or iron starvation, expression of *cwlM* was not significantly altered; however, the intracellular concentration of CwlM protein was ∼2-fold lower in *Ms*-IRE_WT_ compared with the untreated cultures (Table 1; Figure S3A). This response was abolished when the *cwlM*_*Mt*_ IRE was disrupted (Table 1; Figure S3B). Additional quantification of CwlM protein levels at 30 min (Figures 4A and S3C) and 120 min (Figures 4B and S3C) showed similar patterns, suggesting that oxidative stress and iron starvation decreased total CwlM protein in *Ms*-IRE_WT_ but not in *Ms*-IRE5 (Figure S4A). Thus, although the IRE sequence begins 41 bases into the *cwlM* coding region, it appears that its function is the same as that of IREs located in the 5′ UTR (i.e., to inhibit translation) (Goss and Theil, 2011).

**Table 1:** Changes in Expression of *cwlM* Gene and Relative Abundance of CwlM Protein in *M. smegmatis* Cultures Expressing Wild-Type (WT) and Mutated Versions (IRE5) of *cwlM* When Subjected to Stressful Conditions

Treatment^a^	*cwlM* Expression Fold Difference (qRT-PCR)		CwlM Relative Abundance (Western Blot Analysis)	
	WT	IRE5	WT	IRE5
Pristinamycin	4.78 ± 1.84	4.84 ± 0.98	1.58 ± 0.71	1.92 ± 0.44
NO	3.24 ± 0.77	3.22 ± 0.2	1.25 ± 0.75	1.11 ± 0.61
ROS	1.04 ± 0.22	0.97 ± 0.10	0.60 ± 0.0.17	1.05 ± 0.50
BIP	0.97 ± 0.09	0.91 ± 0.3	0.57 ± 0.30	1.11 ± 0.34
ROS+NO	2.67 ± 0.85	3.03 ± 0.67	1.34 ± 0.41	1.62 ± 0.83

a Bacteria were incubated under the indicated conditions for 60 min before isolation of RNA (qRT-PCR) or preparation cell lysates for western blot analysis. Fold difference was calculated relative to untreated bacteria as described in STAR Methods. Mean values ± STDV (n = 3) shown for qRT-PCR data and mean values ± 95% confidence intervals (n = 2) are shown for western blot analyses. See also Figure S3 and Table S1.

As the ROS+NO combination stress had a profound effect on viability of *Ms*-IRE5, the amount of CwlM protein in stressed and untreated cultures of *Ms*-IRE_WT_ and *Ms*-IRE5 was monitored for up to 6 h (Figures 4C and S4B; Table S1). The amounts of CwlM protein in the stressed cultures relative to those in the unstressed cultures from two independent experiments are shown in Figure 4C. After 2 h exposure, both *Ms*-IRE_WT_ and *Ms*-IRE5 treated cultures showed no substantial difference in CwlM abundance compared with untreated control, presumably as a result of two conflicting processes affecting CwlM amounts: transcriptional upregulation of *cwlM* by the NO treatment (Table 1) and downregulation of CwlM translation due to the ROS treatment (Figures 4A and 4B). Examination of the data from individual experiments suggested that there was variation between biological replicates in the balance between these antagonistic responses: downregulation of CwlM protein was evident earlier for *Ms*-IRE_WT_ replicate 1 compared with replicate 2 (Table S1). Nevertheless, after 6 h of exposure to ROS+NO, both *Ms*-IRE_WT_ cultures exhibited decreased CwlM abundance (Figure 4C, WT blue bars, and Figure S4B). In contrast, the *Ms*-IRE5 cultures exhibited the opposite response, such that in the 6 h samples, the relative amount of CwlM protein was greater than it was 2 h after exposure to ROS+NO (Figure 4C, IRE5 red bars, and Figure S4B). Thus, the difference between the CwlM protein present in the ROS+NO stressed *Ms*-IRE_WT_ cultures compared with the ROS+NO stressed *Ms*-IRE5 cultures increased during the course of experiments.

It is worth noting that the nucleotide changes necessary to disrupt the *cwlM* IRE while retaining the primary structure of the CwlM protein required the replacement of four codons (GGC, CGC, GCC, and GTC) that are used more often in highly expressed genes with codons that are less abundant in such genes (Andersson and Sharp, 1996). Nevertheless, the replacement codons (GGG, CGG, GCA, and GTG) did not lower the abundance of the CwlM protein (Figures S3–S5, untreated samples).

Together with the phenotypic studies (Figures 2 and 3), these data suggest that in response to combined ROS/NO stress, the abundance of the CwlM protein initially increases, probably because of an increase in *cwlM* mRNA levels promoted by NO (Table 1), and then decreases consistent with the proposed post-transcriptional repression mediated by apo-AcnA (Figure 4). Without AcnA-IRE-mediated downregulation (i.e., *Ms*-IRE5 strain), elevated levels of CwlM are sustained (Figure 4) and appear to be detrimental, resulting in severe attenuation of growth under oxidative and nitrosative stress conditions (Figures 2H, 3I, and 3J).

Recall that CwlM is a client for the protein kinase PknB and that it performs different regulatory roles depending on its phosphorylation state; namely, non-phosphorylated CwlM interacts with MurJ, presumably affecting the export of peptidoglycan precursors (Turapov et al., 2018), and phosphorylated CwlM (CwlM∼P) interacts with MurA to increase peptidoglycan precursor synthesis (Boutte et al., 2016). Antibodies specific for non-phosphorylated CwlM and CwlM∼P (Figures S5A and S5B) showed that after 6 h exposure to ROS+NO stress, disruption of the *cwlM* IRE (*Ms*-IRE5) resulted in an ∼4.6-fold increase of non-phosphorylated CwlM compared with *Ms*-IRE_WT_ (Figure 4C, gray bars). This suggested that a sustained presence of elevated levels of non-phosphorylated CwlM, as a result of disrupting the IRE, is detrimental when the bacteria are challenged by combined oxidative and nitrosative stresses. In support of this interpretation, ectopically increasing *cwlM* expression by addition of pristinamycin to cultures of the *Ms cwlM*-CM/*cwlM*_*Mt*_ led to higher amounts of CwlM protein (Table 1). Whereas *Ms cwlM*-CM/*cwlM*_*Mt*_ cultures were able to recover after exposure to ROS+NO stress (Figure 2H), the overexpression of CwlM, induced by addition of pristinamycin, resulted in sensitivity to this stress combination (Figure 4D). This is consistent with the notion that failure to downregulate the CwlM protein in response to ROS+NO, either by disabling the *cwlM* IRE or by ectopic overexpression of *cwlM*, prevents recovery from the effects of these stresses. The toxicity of the phosphoablative CwlM variant speaks to the potential detrimental effects of increased levels of non-phosphorylated CwlM (Turapov et al., 2018). It is suggested that optimal levels of CwlM∼P and non-phosphorylated CwlM protein are maintained through a complex regulatory system, which includes the *cwlM* IRE, in response to host-related stresses, facilitating mycobacterial survival and recovery, consistent with the growth phenotypes reported above (Figures 2 and 3).

### Disruption of the *cwlM* IRE Compromises *M. tuberculosis* H37Rv Growth in THP-1 Macrophages

**Figure 5:**
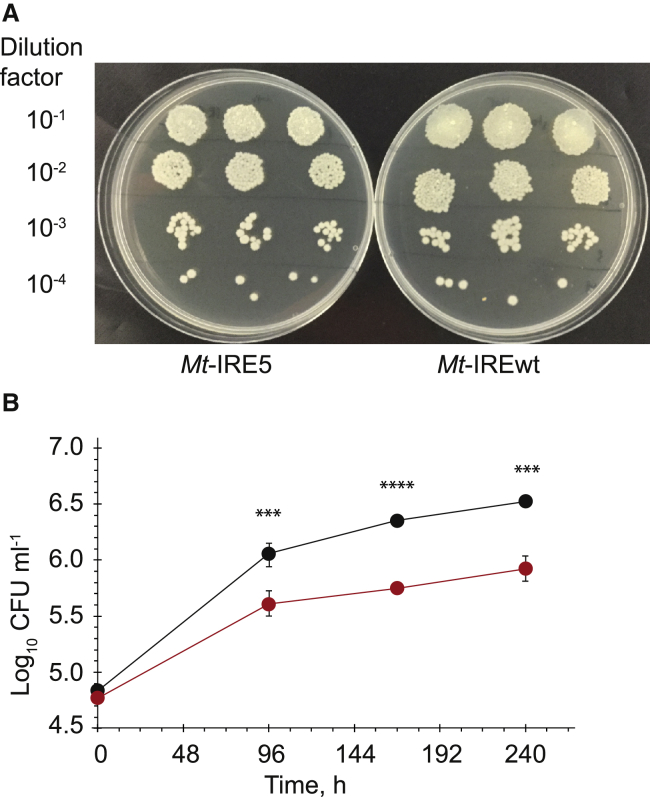
Disruption of the *cwlM* IRE Impairs *M. tuberculosis* Growth in THP-1 Macrophages (A) Cultures of *M. tuberculosis cwlM-*CM complemented with either wild-type *cwlM*_*Mt*_ (IRE_WT_, right image) or *cwlM*_*Mt*_-IRE5 (IRE5, left image) were diluted as indicated on the left of the panel and spotted onto Middlebrook 7H10 plates and incubated at 37°C for 6 weeks. The growth pattern showed that complementation with *cwlM*_*Mt*_-IRE5 did not cause a general growth defect *in vitro*. (B) *Mycobacterium tuberculosis cwlM* conditional mutant complemented with either wild-type *cwlM* (*Mt* IRE_WT_) or *cwlM* with the disrupted IRE (*Mt* IRE5) was used to infect PMA treated THP-1 cells. Samples for CFU measurements were taken after 0, 4, 7, and 10 days post-infection; data are presented as mean ± SEM (n = 6). CFU counts of *Mt* IRE_WT_ (black circles) were significantly different from *Mt*-IRE5 (red circles) counts (^∗∗∗^p < 0.001 and ^∗∗∗∗^p < 0.0001, unpaired t test).

The experiments described above suggested that AcnA-mediated regulation of *cwlM* is an important component of the mycobacterial response to host-associated stresses. A *M. tuberculosis* H37Rv *cwlM* conditional mutant (*Mt cwlM*-CM) that could be complemented by either native *cwlM*_*Mt*_ or *cwlM*_*Mt*_-IRE5 was created (referred to as *Mt*-IRE_WT_ and *Mt*-IRE5, respectively). Growth of both strains was indistinguishable *in vitro*, indicating that disrupting the *cwlM* IRE did not result in a general growth defect (Figure 5A). The THP-1 cell line was treated with phorbol 12-myristate 13-acetate (PMA) to differentiate monocytes into macrophages. These were subsequently infected with *Mt*-IRE_WT_ or *Mt*-IRE5 at a low multiplicity of infection (0.4) to mimic encounters between *M. tuberculosis* and macrophages during initial infection. A significant growth defect of the bacteria was evident when the *cwlM* IRE was disrupted: intracellular growth of *Mt*-IRE_WT_ resulted in a rapid, 17-fold increase in CFU counts within the first 4 days of infection, reaching ∼5 × 10^6^ CFU ml^−1^ after 10 days, while growth of *Mt*-IRE5 resulted in ∼3-fold lower CFU counts on day 4 compared to *Mt*-IRE_WT_ and <1 × 10^6^ CFU ml^−1^ at the end of experiment, suggesting that regulation mediated through the *cwlM* IRE contributes to the ability of *M. tuberculosis* to replicate in THP-1 macrophages (Figure 5B). Furthermore, at day 10 the THP-1 macrophages infected with *Mt*-IRE_WT_ began to detach from the plastic surface and exhibited aggregation behavior, whereas those infected with *Mt*-IRE5 did not, suggesting that this phenotype could be related to high bacterial loads as previously reported (Schaaf et al., 2016).

## Discussion

The central metabolic intermediate α-ketoglutarate has been shown to be critical for growth and viability of *Caulobacter crescentus* by linking the operation of the Krebs cycle and peptidoglycan synthesis (Irnov et al., 2017). In *M. tuberculosis*, central metabolism has a key role in persistence and pathogenesis, enabling growth in various nutritional niches (Ehrt et al., 2018). Shut-down and resumption of *M. tuberculosis* peptidoglycan synthesis is characteristic of TB pathogenesis (e.g., during entry into and emergence from dormancy), associated with latent infection in response to the effectiveness of the host immune system. Hence, transition of *M. tuberculosis* to hypoxic conditions reprograms central metabolism to enter survival mode, which includes use of mycolyl-glycolipids as a source of energy and accumulation of UDP-*N*-acetylglucosamine enoyl-pyruvate, an essential peptidoglycan precursor, via a non-MurA and ATP-consuming mechanism (Eoh et al., 2017). However, very little is known about adaptation of peptidoglycan precursor synthesis in mycobacteria in response to host-related stresses (Maitra et al., 2019). The experimental data presented in this study indicate the imperative to couple peptidoglycan synthesis to central metabolic activity when mycobacteria are challenged by combinations of host-associated stresses. This is achieved by a complex regulatory system that controls the function of the essential peptidoglycan synthesis regulator, CwlM. Multi-level regulation operates to increase *cwlM* mRNA under nitrosative stress and decrease CwlM protein abundance in response to oxidative stress/iron starvation (presumably by apo-AcnA binding at the *cwlM* IRE) and post-translational modification (CwlM phosphorylation by PknB).

The global response of *M. tuberculosis* to nitrosative stress involves reprogramming gene expression driven by the necessity to replace damaged (mainly iron-sulfur-containing) proteins (Cortes et al., 2017). The mechanism underlying the NO-mediated upregulation of *cwlM* expression observed here is unknown, although it might be caused by increased expression of *sigE* during NO treatment (Cortes et al., 2017). Nevertheless, it appears that this response is beneficial for survival (Figure 2E).

Here it is shown that an IRE in the *cwlM* transcript is critical for mycobacterial survival upon exposure to combined stresses, such as might be encountered within a host. Hence, combining oxidative stress with iron starvation or NO treatment resulted in a dramatic drop in *Ms*-IRE5 counts compared with *Ms*-IRE_WT_, presumably as a result of the combined damaging effect of the stressors on cellular components and dysregulated expression of CwlM protein (Figures 2 and 3).

In growing mycobacteria, CwlM protein is found in two forms: cytoplasmic CwlM∼P and membrane-associated non-phosphorylated CwlM. Overabundance of non-phosphorylated CwlM is toxic for mycobacteria (Turapov et al., 2018), and hence the balance between phosphorylated and non-phosphorylated forms is maintained by PknB-mediated phosphorylation and possibly by sequestration of CwlM∼P by the FhaA protein (Turapov et al., 2018). However, the data presented here suggest that upon exposure to host-related stresses, mycobacteria are able to adapt and maintain a “balanced” population of CwlM through the operation of an IRE-mediated post-transcriptional mechanism. This is manifest upon disruption of the *cwlM* IRE, which resulted in increased abundance of toxic non-phosphorylated CwlM protein when mycobacteria were exposed to combinations of iron starvation and oxidative and nitrosative stresses (Figure 4C), leading to impaired survival of *M. smegmatis in vitro* and attenuation of *M. tuberculosis* in THP-1 macrophages (Figure 5B).

**Figure 6:**
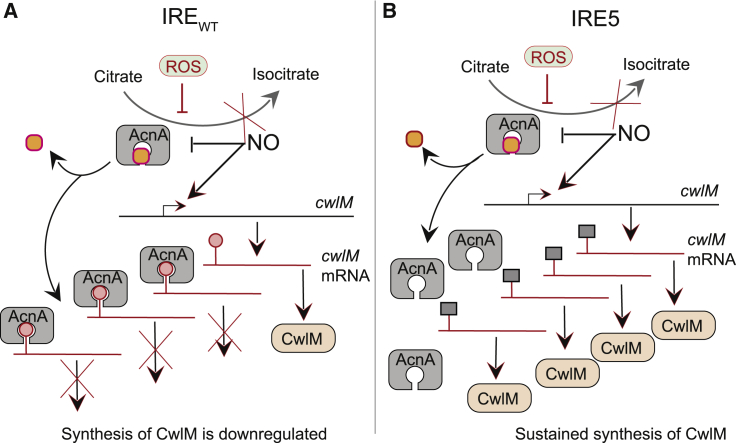
The Role of AcnA-Mediated Post-transcriptional Regulation of Mycobacterial CwlM in Response to Combined Oxidative and Nitrosative Stresses In unstressed bacteria, aconitase (AcnA; gray rounded square) acquires its active-site [4Fe-4S] cluster (orange circle) and acts as an essential enzyme converting citrate to isocitrate in the Krebs and glyoxylate cycles. The *cwlM* mRNA (red lines) is synthesized, and the CwlM protein (gray rounded rectangle) is produced. (A) Upon exposure of wild-type mycobacteria to combined nitrosative (NO) and oxidative (ROS) stress, NO increases the relative abundance of the *cwlM* transcript (Table 1), and ROS disassemble the AcnA iron-sulfur cluster (Figure 1E). This response halts central metabolism (Krebs cycle) by abolishing AcnA catalytic activity (red X; Figure 1E) and generates the apo form of AcnA that is capable of binding the *cwlM* IRE (red lollipop-shaped structures; Figure 1F). Binding of apo-AcnA to the *cwlM* IRE downregulates synthesis of CwlM protein (red X; Figure 4) until the stress is relieved, at which point the AcnA iron-sulfur cluster is repaired and growth resumes. (B) Removal of the *cwlM* IRE (IRE5; gray square) abolishes binding of apo-AcnA to the *cwlM* transcript upon exposure to ROS+NO stress and CwlM protein synthesis is sustained (Figure 4) in the absence of central metabolic activity. The failure to inhibit *cwlM* translation results in increased levels of toxic, non-phosphorylated CwlM protein (Figure 4C), manifest as an inability to recover from combined ROS+NO stress and impaired growth in THP-1 macrophages (Figures 2H, 2J, 2K, and 5B).

The degree of attenuation of *Mt*-IRE5 in THP-1-derived macrophages was less severe than anticipated on the basis of the growth defects when *Ms*-IRE5 was exposed to combinations of RNS, ROS, and iron starvation *in vitro* (Figure 2). However, *M. tuberculosis* is well adapted to colonize macrophages, possessing several dedicated sensor-regulator systems for responding to the ROS, RNS, and iron starvation stimuli investigated here (e.g., DosS/T/R, WhiB-like proteins, Cmr, and IdeR) (Cumming et al., 2014; Pandey and Rodriguez, 2014; Kudhair et al., 2017; Smith et al., 2017). Alongside these responses, systems have evolved to facilitate survival of *M. tuberculosis* in alveolar macrophages by subverting normal functions to convert potentially bacteriocidal macrophages into permissive niches (Queval et al., 2017). Here it is shown that the IRE-like sequence of the essential peptidoglycan regulator, CwlM, is a previously unrecognized contributor to the survival of mycobacteria under stress conditions. It is speculated that apo-AcnA binding to the IRE impairs *cwlM* translation, preventing futile production and export of peptidoglycan precursors when the mycobacteria are unable to grow under the stresses imposed by combinations of RNS and ROS (Figure 6). If left unchecked, such futile synthesis would ultimately exhaust mycobacterial resources. To ensure that this does not occur, when growth is inhibited by RNS, ROS, and/or iron sequestration, the AcnA iron-sulfur cluster “fuse” is “blown,” halting central metabolism. The resulting apo-AcnA protein binds at the *cwlM* IRE to repress peptidoglycan synthesis until the mycobacteria adapt to the new conditions (via detoxification of stressors and repair/replacement of damaged cell components). Upon restoration of redox balance and iron homeostasis, AcnA reacquires its iron-sulfur cluster, resulting in disengagement from the *cwlM* IRE, permitting immediate initiation of CwlM translation simultaneously restoring peptidoglycan synthesis and mycobacterial growth. Although this interpretation is consistent with the available experimental data, one of the limitations of the present study is the absence of direct evidence of apo-AcnA binding to *cwlM* mRNA *in vivo* and that it is this interaction that is responsible for the stress-induced phenotypes reported here and not some unknown transcriptional/translational regulation. Future work might be directed toward creation of an AcnA variant that retains catalytic activity but lacks the ability to bind IREs, to show that strains expressing such a variant were hypersensitive to challenge by the ROS and NO combination, as observed for the IRE5 mutant. However, because AcnA and CwlM are both essential, these experiments will be challenging and perhaps difficult to interpret, at least in part because the *cwlM* IRE is not unique. Apo-AcnA also binds an IRE located within the 5′ UTR of the *ideR* transcript and thus, as well as repressing peptidoglycan synthesis and export, likely derepresses the expression of iron acquisition systems to maintain iron homeostasis by inhibiting translation of *ideR* (Rodriguez et al., 2002; Banerjee et al., 2007). Furthermore, although the focus of the present work has been on the post-transcriptional regulation of CwlM, it remains possible that the *cwlM* IRE plays a role in regulating TrxB2/TrxC, as originally proposed, if a *trxB2-trxC-cwlM* transcript from the *trxB2* promoter is produced under some conditions (Figure 1A; Banerjee et al., 2007). TrxB2 is essential for growth of *M. tuberculosis in vitro* and *in vivo*, and depletion of TrxB2 caused lysis of *M. tuberculosis* in laboratory cultures (Lin et al., 2016). As a regulator of peptidoglycan synthesis, dysregulation of CwlM could induce cell lysis, and it is tempting to speculate that both TrxB2 and CwlM contribute to the decreases in CFU and OD_600_ values observed upon disruption of the *cwlM* IRE (Figures 2H and 3J). Further work will be required to investigate this possibility, but it is noted that *trxB2-trxC* were not required to complement the *cwlM* conditional mutants used here.

It has been suggested that mRNA molecules could emerge as prime targets for small-molecule drugs (Warner et al., 2018). Here a potential vulnerability of mycobacteria to drugs targeting IREs has been revealed. When challenged by macrophages, disruption of the *cwlM* IRE impairs replication of *M. tuberculosis*, and hence developing small-molecule drugs that target the *cwlM* and *ideR* IREs could open new opportunities to control TB infections.

## STAR★Methods

### Key Resources Table

**Table:** 

REAGENT or RESOURCE	SOURCE	IDENTIFIER
**Antibodies**		

Rabbit serum raised against *Escherichia coli* AcnA	Prodromou et al.,1991	N/A
Custom polyclonal CwlM antibodies raised in rabbit	Turapov et al., 2018	N/A
Custom polyclonal antibodies raised against GKNDRPT-phospho-GT in rabbit (anti-T382-P)	Turapov et al., 2018	N/A
Custom polyclonal antibodies raised against GKNDRPTGT in rabbit (anti-T382)	Turapov et al., 2018	N/A
Anti-rabbit IgG, HRP-linked antibody	ThermoFisher Scientific	Cat# 32460; PRID: AB_1185567
Rabbit serum raised against *Escherichia coli* AcnA	Prodromou et al.,1991	N/A

**Bacterial Strains**		

*Escherichia coli* BL21 (λDE3)	Laboratory collection	N/A
*Escherichia coli* BL21 (λDE3) pET28a.*acn*	This work	N/A
*Escherichia coli* DH5α	Laboratory collection	N/A
*Escherichia coli* DH5α pGEM-3Zf.IRE(WT)	This work	N/A
*Escherichia coli* DH5α pGEM-3Zf.IRE(IRE5)	This work	N/A
*Escherichia coli* DH5α pMV306.*cwlM*_*Mt*_ (WT)	This work	N/A
*Escherichia coli* DH5α pMV306.*cwlM*_*Mt*_ (IRE5)	This work	N/A
*Escherichia coli* XL1-Blue Supercompetent cells	Agilent	Cat# 200236
*Mycobacterium smegmatis* mc^2^155	Laboratory stock	ATCC 700084
*Mycobacterium smegmatis* conditional mutant *cwlM*	This work	N/A
*Mycobacterium smegmatis* conditional mutant *cwlM* pMV306::*cwlM*_*Mt*_ (WT)	This work	N/A
*Mycobacterium tuberculosis* H37Rv	Laboratory stock	ATCC 27294
*Mycobacterium tuberculosis* H37Rv conditional mutant *cwlM* pMV306::*cwlM*_*Mt*_ (WT)	Turapov et al., 2018	N/A
*Mycobacterium tuberculosis* H37Rv conditional mutant *cwlM* pMV306::*cwlM*_*Mt*_ (IRE5)	This work	N/A

**Chemicals, Peptides, and Recombinant Proteins**		

BD Difco Middlebrook 7H9 Broth	ThermoFisher Scientific	Cat# DF0713-17-9
BD Difco Middlebrook 7H10 Agar	ThermoFisher Scientific	Cat# DF0627-17-4
cOmplete Ultra Tablets Protease Cocktail	Sigma-Aldrich	05892970001 ROCHE
Hygromycin	ThermoFisher Scientific	Cat# 10687010
*Mycobacterium tuberculosis* AcnA protein with a N-terminal hexa-His tag	This work	N/A
Pristinamycin	Molcan	Cat# PSM01A-100

**Critical Commercial Assays**		

Pierce Ultra 1-Step TMB-Blotting Solution	ThermoFisher Scientific	Cat# 37574
QuikChange II	Agilent	Cat# 200523
SensiFAST SYBR Lo-ROX One-Step Kit	Bioline	Cat# BIO-94005

**Deposited Data**		

This study did not generate any unique datasets or code	N/A	N/A

**Experimental Models: Cell Lines**		

THP-1	Laboratory stock	ATCC TIB-202

**Oligonucleotides**		

Oligonucleotides were custom synthesized (details provided in Table S2)	Sigma Aldrich	N/A

**Recombinant DNA**		

pET28a.*acn*	This work	N/A
pGEM-3Zf.IRE(WT)	This work	N/A
pGEM-3Zf.IRE(IRE5)	This work	N/A
pMV306.*cwlM*_*Mt*_ (WT)	This work	N/A
pAZI9479::*cwlM*_*Ms*_	This work	N/A
pAZI9479::*cwlM*_*Mt*_	Turapov et al., 2018	

**Software and Algorithms**		

ImageJ	Schneider et al., 2012	https://imagej.nih.gov/ij/ PRID:SCR_003070

### Resource Availability

#### Lead contact

Further information and requests for reagents and resources should be directed to Galina V. Mukamolova (gvm4@leicester.ac.uk).

#### Materials availability

Bacterial strains and plasmids are available on request.

#### Data and code availability

This study did not generate any unique datasets or code.

### Experimental Model and Subject Details

#### Bacterial strains, plasmids, and culture conditions

Bacterial strains, plasmids, and oligonucleotides used in this research are listed in Key Resources Table and Table S2. Authenticity of all plasmids was confirmed by DNA sequencing. *Escherichia coli* BL21 (λDE3) pGS2532 (JRG6822) was used for overproduction of recombinant *M. tuberculosis* aconitase (AcnA). The expression plasmid pGS2532 has the *M. tuberculosis* H37Rv *acn* open reading frame in pET28a.

*Mycobacterium smegmatis* mc^2^155 cultures were grown in Middlebrook 7H9 broth or 7H10 agar complete medium (BD Difco) supplemented with Middlebrook ADC (2.5 g BSA fraction V, 1 g dextrose, 1.5 mg catalase in 50 mL distilled water) and 0.05% Tween-80 at 37°C with shaking at 200 rpm.

The *M. smegmatis cwlM* conditional mutant (*Ms cwlM*-CM) was created by amplifying a 750 bp DNA 5′ fragment of the *msmeg_6935* gene using primers msmeg_6935F and msmeg_6935R and Platinum™ *Taq* DNA polymerase. This fragment was cloned into NcoI and SphI sites of the pAZI9479 plasmid. After sequencing, 1 μg of the construct was electroporated into *M. smegmatis* and transformants selected on 7H10 agar containing hygromycin (50 μg ml^-1^) and pristinamycin (2 μg ml^-1^). The previously described *Mt cwlM*-CM was used for THP-1 macrophage infection (Turapov et al., 2018). For complementation of *Ms cwlM*-CM and *Mt cwlM*-CM mutants the pMV306::*cwlM*_*Mt*_ plasmid containing a coding sequence of *Rv3915* (*cwlM*) with a 200 bp-upstream region was used as previously described (Turapov et al., 2018).

Site-directed mutagenesis (SDM) was carried out using the QuikChange II Sire-Directed Mutagenesis Kit (Stratagene). First an intermediary IRE mutant gene was prepared using the plasmid pMV306::*cwlM*_*Mt*_ as the template and the oligonucleotides PB01 and PB02. In a second round of mutagenesis the oligonucleotides PB03 and PB04 and the intermediate (the template) were used to generate the plasmid pMV306.*cwlM*_*Mt*_ IRE5. Mutations were confirmed by purifying and sequencing the plasmids.

To determine appropriate concentrations of stress reagents for phenotypic studies (Figures 2 and 3), strains of *M. smegmatis* were grown in 7H9 broth to OD_600_ ∼0.8, then diluted 1:1000 in 50 ml fresh broth containing plumbagin, spermine NONOate or 2,2′-bipyridyl at 1 μM and increasing in 2-fold increments to 256 μM. At the concentration where no growth was observed after 96 h, the procedure was repeated using concentrations of stress reagent at 25 μM intervals below the inhibitory concentration to more precisely determine the minimum inhibitory concentration. Strains of *M. smegmatis cwlM*-CM were then grown in 7H9 broth to OD_600_ ∼0.8, then diluted 1:1000 in 50 ml fresh broth containing the stress reagents at sub-inhibitory concentrations (plumbagin [75 μM], spermine NONOate [200 μM] or 2,2′-bipyridyl [100 μM]). Broths were incubated at 37°C with shaking at 200 rpm for 96 h. Samples of broth were taken every 24 h, diluted 10-fold in series and plated on 7H10 agar. Plates were incubated at 37°C for 48 h and colonies were counted. For each assessment three experiments were performed.

To determine the effects of the stress reagents on turbid *M. smegmatis cwlM*-CM strains (Figure 3), cultures were grown in 7H9 broth to OD_600_ ∼0.6, before adding the stress reagents at the concentrations indicated above directly to the cultures. Cultures were either aliquoted onto a 96-well plate and incubated at 37°C with shaking at 200 rpm in a plate reader with absorbance at 600 nm recorded every 30 min for 12 h, or the cultures were incubated at 37°C and the colony-forming units estimated every 2 h for 12 h. For each assessment two experiments were performed.

For most probable number (MPN) estimations, cultures were diluted 1:10 in four tubes of 5 ml fresh 7H9 broth and incubated at 37°C for up to 5 days. Turbid broths were diluted in series to a maximum dilution of 1:10^8^. Values for MPN were then calculated (Jarvis et al., 2010). For each assessment three experiments were performed.

### Method Details

#### Preparation of competent cells

Cultures of *E. coli* DH5α and *E. coli* BL21 (λDE3) were grown to OD_600_ ∼0.4 in LB broth (50 ml) and transferred to 50 mL Falcon tubes in an ice water bath for 15 min. Cells were collected by centrifugation at 4,000 x *g* for 5 min at 4°C in a pre-cooled centrifuge. Supernatants were discarded and the pellets re-suspended in ice-cold Milli-Q water (5 ml). Centrifugation and re-suspension were repeated twice, with the final re-suspension in ice-cold Milli-Q water containing 10% (v/v) glycerol (250 μl). The suspension was aliquoted (50 μl) into pre-cooled tubes and frozen at −80°C.

Cultures of *M. smegmatis*-conditional mutants were grown to OD_600_ ∼1.0 in 7H9 broth (100 ml) containing hygromycin (50 μg ml^-1^) and pristinamycin (2 μg ml^-1^). Cultures were dispensed into 50 mL Falcon tubes and incubated on ice for 30 min, before centrifugation at 4,000 x *g* for 15 min at 4°C in a pre-cooled centrifuge. Supernatants were discarded and the pellets were re-suspended in 35 mL ice-cold glycerol (10% [v/v]). Centrifugation and re-suspension were repeated twice, with the final re-suspension in 3 mL ice-cold 10% (v/v) glycerol. The suspensions were aliquoted (400 μl) into pre-cooled electroporation cuvettes and used immediately.

#### Transformation of competent cells

Aliquots of electrocompetent *E. coli* cells were thawed on ice, mixed with plasmid DNA (500 ng) and transferred to 1 mm electroporation cuvettes. Cuvettes were pulsed at 1800 V. Cells were diluted with LB (1 ml), transferred to 1.5 ml tubes and incubated at 37°C for 1 h. Samples (50 μl) were spread on LB agar containing appropriate antibiotics and incubated overnight at 37°C to allow growth of transformants. For electroporation of *M. smegmatis* conditional mutants, aliquots (400 μl) of electrocompetent cells were pulsed with plasmid DNA (50-100 ng) at 2500 V. Pulsed cells were diluted with 7H9 broth (3 ml) and incubated at 37°C for 3 h before plating on 7H10 agar containing appropriate antibiotics with and without pristinamycin. Plates were incubated at 37°C for 3 days to allow growth of bacteria.

#### THP-1 infection experiments

The monocytic cell line, THP-1 (ATCC® TIB-202) was maintained in RPMI 1640 supplemented with fetal bovine serum (10% [v/v]), L-glutamine (2 mM) and sodium pyruvate (1 mM) at 37°C in the presence of 5% CO_2_ atmosphere. Macrophages were differentiated using 100 ng/ml phorbol myristate acetate (PMA) for 24 h followed by a 24 h resting period. Macrophages (∼2 × 10^5^) were infected with *M. tuberculosis* at multiplicity of infection 0.4 for 3 h, followed by treatment with amikacin at 200 μg ml^-1^ for 1 h. Amikacin was removed by washing and infected macrophages were incubated at 37°C under 5% CO_2_ for up to 10 d. RPMI medium was replaced every 3 days. For determination of CFU counts macrophages were lysed in SDS (0.006% [w/v]); lysates were serially diluted in 7H9 medium and plated on 7H10 agar. Plates were incubated at 37°C under 5% CO_2_ for up to 6 weeks. Three biological replicates were used for each time point.

#### Overproduction and purification of AcnA

For isolation of the overproduced recombinant AcnA, *E. coli* BL21 (λDE3) pET28a.*acn* was grown to OD_600_ ∼0.8 and diluted 1:1000 into 1 l of Terrific broth (Tryptone, 12 g l^-1^; Yeast Extract, 24 g l^-1^; KH_2_PO_4_, 17 mM; K_2_HPO_4_, 74 mM) containing kanamycin (50 μg ml^-1^) and isopropyl β-D-1-thiogalactopyranoside (120 μg ml^-1^). Bacteria were cultured overnight at 37°C with agitation. Cells were collected by centrifugation at 4,000 x *g* for 30 min at 4°C. The cell pellet was re-suspended in lysis buffer (Tris-HCl [50 mM; pH 7.5], NaCl [200 mM], phenylmethylsulfonyl fluoride [1 mM] and cOmplete Mini EDTA-free protease inhibitors [Sigma-Aldrich]) in a 1:3 ratio of cell weight to buffer volume. Suspensions were sonicated with 10 pulses at an amplitude of 15 μm, each pulse lasting 10 s with a 20 s rest on ice between pulses. Extracts were clarified by centrifugation at 16,000 x *g* for 30 min at 4°C.

The His-tagged AcnA protein was purified by nickel affinity chromatography using a GE Healthcare HiTrap Chelating HP column. Columns were primed with nickel by loading three column volumes of NiSO_4_ (100 mM). Columns were washed with one column volume of binding buffer (NaH_2_PO_4_ [50 mM], NaCl [300 mM], imidazole [20 mM], pH 8.0) and then the clarified bacterial lysate was loaded onto the column. After the protein was loaded, columns were washed with three column volumes of binding buffer and then protein was eluted in three column volumes of elution buffer (NaH_2_PO_4_ [50 mM], NaCl [300 mM], imidazole [270 mM], pH 8.0). Fractions (0.5 ml) were collected and samples of each elution fraction were examined by SDS-PAGE to evaluate the purity of AcnA. AcnA-rich fractions were pooled and dialyzed with Tris-sodium-glycerol (TSG) buffer (Tris-HCl [20 mM], NaCl [100 mM], glycerol [3% (v/v)], pH 8.0) for 16 h under anaerobic conditions using cellulose dialysis tubing with a 14,000 Da molecular weight cut-off.

#### Measurement of protein concentration

The concentration of AcnA was determined using the Bio-Rad protein reagent based on the method of Bradford with bovine serum albumin as the standard (Bradford, 1976).

#### Measurement of aconitase activity

The activity of AcnA preparations was measured using a coupled spectrophotometric assay. Reactions were prepared in a volume of 1 ml in quartz cuvettes, containing 100 μl 10x aconitase assay buffer (Tris-HCl [200 mM, pH 8.0], NaCl [1 M], MgCl_2_ [100 mM], NADP^+^ [10 mM], isocitrate dehydrogenase [3 Units, where 1 Unit is defined as the amount of protein that will convert 1 μmol isocitrate to α-ketoglutarate per min at pH 8.0 and 37°C), a desired amount of AcnA, and distilled water up to a total volume of 950 μl. The reaction was initiated by addition of citrate (50 μl; 100 mM). After mixing, absorbance at 340 nm was recorded for 15 min at 37°C. Aconitase specific activities were calculated from the initial rates of change in absorbance at 340 nm using the millimolar extinction coefficient of 6.22 mM^-1^ cm^-1^ for NADPH.

#### Sensitivity of AcnA to stress reagents

Spermine NONOate and 2,2' bipyridyl were added to holo-aconitase at concentrations of 200 μM and 100 μM, respectively. To generate ROS *in vitro*, a combination of hypoxanthine (6 mM) and xanthine oxidase (0.1 U ml^-1^ where 1 U converts 1 μmol xanthine to uric acid min^-1^ at pH 8.0 and 37°C) was added to holo-AcnA. The treated AcnA was incubated at 37°C for 3 h and then dialyzed with fresh TSG buffer (see above). Iron concentration and aconitase activities were recorded before stress and after dialysis. For each assessment three experiments were performed.

#### Preparation of apo- and reconstituted AcnA

The iron chelator, 2,2′-bipyridyl was added to purified and dialyzed AcnA preparations to a final concentration of 100 μM and incubated at room temperature for 1 h. Samples were then dialyzed into TSG buffer (see above) for 16 h under anaerobic conditions.

Purified and dialyzed AcnA was reconstituted by combining equal volumes of the AcnA preparation and 2x reconstitution reagent (Tris-HCl [50 mM], NaCl [200 mM], Fe(NH_4_)_2_(SO_4_)_2_ [1 mM], dithiothreitol [5 mM], pH 8.0) and incubating for 20 min at 25°C under anaerobic conditions. Samples were transferred to anaerobic microcentrifuge tubes and centrifuged at 10,000 x *g* for 10 min. Supernatants were dialyzed against TSG buffer (see above) for 16 h under anaerobic conditions.

#### Iron content of AcnA and UV-visible spectroscopy

The iron content of AcnA was measured by mixing a sample of protein of known concentration (400 μl) with trichloroacetic acid (66 μl, 12% [w/v]) in a 1.5 mL microcentrifuge tube. The mixture was heated to 100°C for 5 min and then centrifuged at 10,000 x *g* for 3 min. The supernatant was transferred to a new tube and mixed with bathophenanthroline disulfonic acid (30 μl 1% [w/v]), 3 M sodium acetate (400 μl), L-ascorbic acid (90 μl 20% [w/v]) and distilled water to a final volume of 1 ml. The mixture was transferred to a cuvette and the absorbance at 535 nm was recorded. The iron concentration of unknown samples was interpolated from a standard graph prepared using standard iron solutions (Sigma-Aldrich). For untreated preparations seven experiments were performed; for treated samples (see above) three experiments were performed.

UV-visible spectra of purified AcnA in TSG buffer (see above) were collected using a Cary Eclipse spectrophotometer. Samples were placed in a quartz cuvette (1 ml) and absorbance was measured between 200 nm and 800 nm.

#### RNA electrophoretic mobility shift assays (EMSA)

DNA oligonucleotide pairs were designed for the native *cwlM* IRE and the variant with 5 base substitutions in the IRE (*cwlM*-IRE5). Pairs of complementary oligonucleotides (500 ng) were mixed in 5 μl annealing buffer (Tris [10 mM], NaCl [50 mM], EDTA [1 mM], pH 7.6) in a microcentrifuge tube. Mixtures were heated to 95°C for 5 min and then allowed to cool to room temperature. The annealed oligonucleotides were then ligated into a pGEM-3Zf plasmid vector in the following reaction: EcoRI-treated plasmid (250 ng), oligonucleotide duplex (750 ng), 10 μl 10 x ligation buffer (Tris-HCl [300 mM], MgCl_2_ [100 mM], dithiothreitol [100 mM], ATP [10 mM], pH 7.6), T4 DNA ligase (50 U, where 1 U converts 1 nmol ^32^P to Norit-absorbable material in 20 min at 37°C) and nuclease-free water to a final volume of 100 μl. The ligation reaction was mixed and incubated at room temperature for 2 h. Electrocompetent *E. coli* DH5α cells were transformed with the ligated plasmid. Plasmid was extracted using the QIAGEN QiaPrep Spin plasmid miniprep kit and the presence of the insert(s) was confirmed by DNA sequencing using the M13F and M13R primers.

For *in vitro* transcription (IVT), purified plasmid was linearized using BamHI. The IVT reaction was performed using the MAXIscript SP6/T7 kit. The reaction mixture for producing radiolabeled RNA was as follows: linear plasmid (1 μg), 10x transcription buffer (Tris-HCl [30 mM], MgCl_2_ [250 mM], dithiothreitol [100 mM], spermidine [20 mM], Triton X-100 [0.1% (v/v)], pH 8.0; 2 μl), ATP (10 mM, 1 μl), GTP (10 mM, 1 μl), UTP (10 mM,1 μl)], [α^32^P]-CTP (800 Ci mmol^-1^, 5 μl), RNase inhibitor (1 μl)], T7 RNA polymerase (2 μl), nuclease-free water to a final volume of 20 μl. The reaction mixture was mixed by vortexing and briefly centrifuged, then incubated at room temperature for 20 min. Reactions were then mixed with TURBO DNase (1 μl) and incubated at 37°C for 15 min. Unlabelled RNA was prepared using CTP (10 mM, 1 μl) in place of the radiolabeled CTP. The RNA products were precipitated by mixing with EDTA (0.5 M, 1 μl, pH 8.0), ammonium acetate (3 M, 82.5 μl) and absolute ethanol (273 μl). Mixtures were incubated at room temperature for 5 min, then centrifuged at 16,000 x *g* for 10 min and supernatants were discarded. The pellets were mixed with ethanol (70% [v/v], 100 μl), centrifuged again, and the supernatants were discarded. The pellets were dissolved in nuclease-free water. The concentration of unlabeled RNA was estimated by absorbance; radiolabeled RNA was quantified by liquid scintillation.

Polyacrylamide gels for EMSA were prepared in volumes of 10 ml: 10x TBE buffer (Tris [1 M, pH 8], boric acid [1 M], EDTA [20 mM], 0.5 ml), acrylamide/bis-acylamide solution (40% [w/v], 37.5:1, 1.25 ml), distilled water (8.14 ml), ammonium persulfate (10% [w/v], 0.1 ml) and TEMED (10 μl). Samples of RNA prepared by IVT were heated at 95°C for 1 min and allowed to cool to room temperature. Binding reactions were prepared in volumes of 20 μl and consisted of 5x Acn-IRE binding buffer (Tris-HCl [50 mM, pH 7.5], NaCl [250 mM], MgCl_2_ [5 mM], EDTA [2.5 mM], dithiothreitol [2.5 mM], glycerol [20% (v/v)], 4 μl), RNA and AcnA in varying concentrations, and nuclease-free water to a final volume of 20 μl. Reactions were incubated at room temperature for 1 h. Before electrophoresis, 6x EMSA loading buffer (Tris-HCl [10 mM], bromophenol blue [0.25% (w/v)], xylene cyanol [0.25% (w/v)], glycerol [30% (v/v)], 4 μl) was added to each reaction. Samples were loaded onto the gel and separated at a constant 100 V with 0.5x TBE running buffer for approximately 40 min. Gels were enclosed in plastic wrap, dried, and exposed to a phosphorimaging plate for visualization. Supershift EMSAs used a polyclonal serum raised in rabbits against *E. coli* AcnA (Prodromou et al., 1991).

#### Quantitative reverse transcription PCR (qRT-PCR)

The *M. smegmatis cwlM*-CM were grown in 7H9 broth to OD_600_ ∼0.6, then stress reagents were added as previously described. After a further 1 h of incubation at 37°C, bacteria were harvested by centrifugation. Cell pellets were weighed and re-suspended in RNAzol (1 ml per 100 mg of pellet). The suspension was added to an aliquot of zirconia beads and homogenized by pulsing 3 times at 6.0 m s^-1^ for 45 s with a rest of 2 min on ice between pulses. Samples were centrifuged to pellet the beads and cell debris, the supernatant was transferred to a new microfuge tube, centrifuged again, and transferred to another clean tube. The RNA was precipitated as described above and the concentration of RNA was estimated by absorbance. All qRT-PCR reactions were performed using the Bioline SensiFAST SYBR Lo-ROX One-Step kit and measured using a Stratagene Mx3005P qPCR machine. Reactions were cycled as follows: 10 min at 45°C; 2 min at 95°C; 40 cycles of 5 s at 95°C, 25 s at 55°C and 20 s at 72°C; 1 min at 95°C; 30 s at 55°C; 30 s at 95°C. Three biological replicates were analyzed, each with three technical replicates, and C_T_ values were recorded for amplification of *cwlM* and *rrsA*. Fold change in *cwlM* was calculated using the ΔΔC_T_ method, with *cwlM* levels normalized against *rrsA*.

#### Western blot analysis of CwlM

Cultures of *Ms cwlM*-CM were grown in 7H9 broth (100 ml) to OD_600_ ∼0.6, then stress reagents were added and cultures were incubated at 37°C for a further 30 min to 6 h. Cells were harvested by centrifugation and re-suspended in 1 ml ice-cold lysis buffer (Tris-HCl [20 mM], NaCl [150 mM], pH7.4) containing Na_3_VO_4_ (1 mM) and protease inhibitors (see above). Samples were kept on ice, before sonication (3 times with a 5 s pulse at 15 μm amplitude followed by 3 min on ice). The sonicator probe was cooled with dry ice prior to each pulse. Lysates were centrifuged at 2500 x *g* for 15 min at 4°C in a pre-cooled microcentrifuge. Supernatants were transferred to ice-cold 1.5 ml low protein binding microcentrifuge tubes and centrifuged again for 5 min. Supernatants were transferred to an ice-cold ultracentrifuge tube and centrifuged at 27,000 x *g* for 1 h at 4°C in a pre-cooled ultracentrifuge. Supernatant was aliquoted into low protein binding tubes and stored at −80°C. Protein content of lysates was measured via Bio-Rad protein assay (Bradford, 1976). Proteins (20 μg) were separated on 8% SDS-PAGE at constant voltage (50 V) for 5 min and then at 250 V for approximately 40 min. Sample lysates were separated alongside HRP-conjugated protein standards.

The polyacrylamide gels were incubated in transfer buffer (Tris [25 mM], glycine [190 mM], methanol [20% (v/v)]) for 15 min, then separated proteins were transferred to a nitrocellulose membrane by sandwiching the gel and membrane together in a cassette submerged in transfer buffer and subjected to a constant 100 V for 1 h. The transfer tank was kept in an ice bath throughout. After transfer, nitrocellulose membranes were washed three times with TBS buffer (Tris [20 mM], NaCl [150 mM], pH 7.5), then incubated for 30 min with gentle agitation in TBS buffer containing milk powder (5% [w/v]). Membranes were then washed three times with TBS buffer containing Tween 20 (0.1% [v/v]), and then incubated overnight at 4°C with gentle agitation in TBS buffer containing milk powder (5% [w/v]) and anti-CwlM rabbit IgG; anti-CwlM antibodies were purified using Protein A Sepharose, anti-CwlM∼P and anti-non-phosphorylated CwlM antibodies were raised against peptides and the antibodies were purified using affinity chromatography (Turapov et al., 2018). After overnight incubation, membranes were washed three times with TBS buffer containing Tween 20 (0.1% [v/v]) and then incubated for 30 min with gentle agitation in TBS buffer containing milk powder (5% [w/v]). Membranes were then washed three times with TBS buffer containing, Tween 20 (0.1% [v/v]) and then incubated for 3 h with gentle agitation at room temperature in TBS buffer containing milk powder (0.5% [w/v]) and HRP-conjugated anti-rabbit goat IgG. After secondary antibody incubation, membranes were washed three times with TBS buffer containing Tween 20 (0.1% [v/v]). Antibody binding was visualized using Pierce Ultra 1-Step TMB-Blotting Solution. Densitometry analysis of blots was performed using ImageJ (Schneider et al., 2012).

### Quantification and Statistical Analysis

Analyses of aconitase activity and iron content (Figure 1E), *cwlM* transcript abundance (Table 1) and bacterial growth data (Figures 2, and 3, and 5) were done in Microsoft Excel, AcnA kinetic parameters (Figure S1A) were calculated using Graphpad Prism version 7.02; n corresponds to the number of replicates. Statistical analysis was done using unpaired t test.

Densitometric analyses of western blots (Figure 4; Tables 1 and S1) were done using ImageJ version 1.51 (Schneider et al., 2012). The CwlM signals detected by western blotting and the total protein detected in the corresponding Coomassie blue-stained gels were measured. The CwlM signal values were then corrected to allow for differences in total protein loading obtained from the Coomassie blue-stained gel measurements. These corrected CwlM values were used to calculate the ratio of the CwlM signals in the treated cultures relative to the corresponding untreated cultures. The individual measurements from independent experiments (Replicate 1 and Replicate 2) are shown in Table S1. The values were averaged and the population standard deviation was determined. The standard error of the mean was obtained (assuming a normal data distribution) and used to calculate the 95% confidence intervals shown in parentheses in Table 1 and as error bars in Figure 4.

## Supplemental Information

Document S1. Figures S1–S5 and Tables S1 and S2

Document S2. Article plus Supplemental Information

## References

[bib1] Alén C., Sonenshein A.L. (1999). *Bacillus subtilis* aconitase is an RNA-binding protein. Proc. Natl. Acad. Sci. U S A.

[bib2] Andersson G.E., Sharp P.M. (1996). Codon usage in the *Mycobacterium tuberculosis* complex. Microbiology (Reading).

[bib3] Artymiuk P.J., Green J. (2006). The double life of aconitase. Structure.

[bib4] Austin C.M., Wang G., Maier R.J. (2015). Aconitase functions as a pleiotropic posttranscriptional regulator in *Helicobacter pylori*. J. Bacteriol..

[bib5] Banerjee S., Nandyala A.K., Raviprasad P., Ahmed N., Hasnain S.E. (2007). Iron-dependent RNA-binding activity of *Mycobacterium tuberculosis* aconitase. J. Bacteriol..

[bib6] Baothman O.A.S., Rolfe M.D., Green J. (2013). Characterization of *Salmonella enterica* serovar Typhimurium aconitase A. Microbiology (Reading).

[bib7] Betts J.C., Lukey P.T., Robb L.C., McAdam R.A., Duncan K. (2002). Evaluation of a nutrient starvation model of *Mycobacterium tuberculosis* persistence by gene and protein expression profiling. Mol. Microbiol..

[bib8] Boutte C.C., Baer C.E., Papavinasasundaram K., Liu W., Chase M.R., Meniche X., Fortune S.M., Sassetti C.M., Ioerger T.R., Rubin E.J. (2016). A cytoplasmic peptidoglycan amidase homologue controls mycobacterial cell wall synthesis. eLife.

[bib9] Bradford M.M. (1976). A rapid and sensitive method for the quantitation of microgram quantities of protein utilizing the principle of protein-dye binding. Anal. Biochem..

[bib10] Cohen-Gonsaud M., Barthe P., Bagnéris C., Henderson B., Ward J., Roumestand C., Keep N.H. (2005). The structure of a resuscitation-promoting factor domain from *Mycobacterium tuberculosis* shows homology to lysozymes. Nat. Struct. Mol. Biol..

[bib11] Cortes T., Schubert O.T., Rose G., Arnvig K.B., Comas I., Aebersold R., Young D.B. (2013). Genome-wide mapping of transcriptional start sites defines an extensive leaderless transcriptome in *Mycobacterium tuberculosis*. Cell Rep..

[bib12] Cortes T., Schubert O.T., Banaei-Esfahani A., Collins B.C., Aebersold R., Young D.B. (2017). Delayed effects of transcriptional responses in *Mycobacterium tuberculosis* exposed to nitric oxide suggest other mechanisms involved in survival. Sci. Rep..

[bib13] Cumming B.M., Lamprecht D.A., Wells R.M., Saini V., Mazorodze J.H., Steyn A.J.C. (2014). The physiology and genetics of oxidative stress in mycobacteria. Microbiol. Spectr..

[bib14] DeJesus M.A., Gerrick E.R., Xu W., Park S.W., Long J.E., Boutte C.C., Rubin E.J., Schnappinger D., Ehrt S., Fortune S.M. (2017). Comprehensive essentiality analysis of the *Mycobacterium tuberculosis* genome via saturating transposon mutagenesis. MBio.

[bib15] Dulberger C.L., Rubin E.J., Boutte C.C. (2020). The mycobacterial cell envelope—a moving target. Nat. Rev. Microbiol..

[bib16] Ehrt S., Schnappinger D., Rhee K.Y. (2018). Metabolic principles of persistence and pathogenicity in *Mycobacterium tuberculosis*. Nat. Rev. Microbiol..

[bib17] Emptage M.H., Kent T.A., Kennedy M.C., Beinert H., Münck E. (1983). Mössbauer and EPR studies of activated aconitase: development of a localized valence state at a subsite of the [4Fe-4S] cluster on binding of citrate. Proc. Natl. Acad. Sci. U S A.

[bib18] Eoh H., Wang Z., Layre E., Rath P., Morris R., Branch Moody D., Rhee K.Y. (2017). Metabolic anticipation in *Mycobacterium tuberculosis*. Nat. Microbiol..

[bib19] Gee C.L., Papavinasasundaram K.G., Blair S.R., Baer C.E., Falick A.M., King D.S., Griffin J.E., Venghatakrishnan H., Zukauskas A., Wei J.R. (2012). A phosphorylated pseudokinase complex controls cell wall synthesis in mycobacteria. Sci. Signal..

[bib20] Goss D.J., Theil E.C. (2011). Iron responsive mRNAs: a family of Fe^2+^ sensitive riboregulators. Acc. Chem. Res..

[bib21] Gruber A.R., Lorenz R., Bernhart S.H., Neuböck R., Hofacker I.L. (2008). The Vienna RNA websuite. Nucleic Acids Res..

[bib22] Gruer M.J., Artymiuk P.J., Guest J.R. (1997). The aconitase family: three structural variations on a common theme. Trends Biochem. Sci..

[bib23] Guirado E., Schlesinger L.S., Kaplan G. (2013). Macrophages in tuberculosis: friend or foe. Semin. Immunopathol..

[bib24] Irnov I., Wang Z., Jannetty N.D., Bustamante J.A., Rhee K.Y., Jacobs-Wagner C. (2017). Crosstalk between the tricarboxylic acid cycle and peptidoglycan synthesis in *Caulobacter crescentus* through the homeostatic control of α-ketoglutarate. PLoS Genet..

[bib25] Jarvis B., Wilrich C., Wilrich P.T. (2010). Reconsideration of the derivation of Most Probable Numbers, their standard deviations, confidence bounds and rarity values. J. Appl. Microbiol..

[bib26] Jordan P.A., Tang Y., Bradbury A.J., Thomson A.J., Guest J.R. (1999). Biochemical and spectroscopic characterization of *Escherichia coli* aconitases (AcnA and AcnB). Biochem. J..

[bib27] Kana B.D., Gordhan B.G., Downing K.J., Sung N., Vostroktunova G., Machowski E.E., Tsenova L., Young M., Kaprelyants A., Kaplan G., Mizrahi V. (2008). The resuscitation-promoting factors of *Mycobacterium tuberculosis* are required for virulence and resuscitation from dormancy but are collectively dispensable for growth *in vitro*. Mol. Microbiol..

[bib28] Kudhair B.K., Hounslow A.M., Rolfe M.D., Crack J.C., Hunt D.M., Buxton R.S., Smith L.J., Le Brun N.E., Williamson M.P., Green J. (2017). Structure of a Wbl protein and implications for NO sensing by *M. tuberculosis*. Nat. Commun..

[bib29] Kurthkoti K., Amin H., Marakalala M.J., Ghanny S., Subbian S., Sakatos A., Livny J., Fortune S.M., Berney M., Rodriguez G.M. (2017). The capacity of *Mycobacterium tuberculosis* to survive iron starvation might enable it to persist in iron-deprived microenvironments of human granulomas. MBio.

[bib30] Lauble H., Kennedy M.C., Beinert H., Stout C.D. (1994). Crystal structures of aconitase with *trans*-aconitate and nitrocitrate bound. J. Mol. Biol..

[bib31] Lin K., O’Brien K.M., Trujillo C., Wang R., Wallach J.B., Schnappinger D., Ehrt S. (2016). *Mycobacterium tuberculosis* thioredoxin reductase is essential for thiol redox homeostasis but plays a minor role in antioxidant defense. PLoS Pathog..

[bib32] Maitra A., Munshi T., Healy J., Martin L.T., Vollmer W., Keep N.H., Bhakta S. (2019). Cell wall peptidoglycan in *Mycobacterium tuberculosis*: an Achilles’ heel for the TB-causing pathogen. FEMS Microbiol. Rev..

[bib33] Manganelli R. (2014). Sigma factors: key molecules in *Mycobacterium tuberculosis* physiology and virulence. Microbiol. Spectr..

[bib34] Michta E., Ding W., Zhu S., Blin K., Ruan H., Wang R., Wohlleben W., Mast Y. (2014). Proteomic approach to reveal the regulatory function of aconitase AcnA in oxidative stress response in the antibiotic producer *Streptomyces viridochromogenes* Tü494. PLoS ONE.

[bib35] Mukamolova G.V., Turapov O.A., Young D.I., Kaprelyants A.S., Kell D.B., Young M. (2002). A family of autocrine growth factors in *Mycobacterium tuberculosis*. Mol. Microbiol..

[bib36] Nambi S., Long J.E., Mishra B.B., Baker R., Murphy K.C., Olive A.J., Nguyen H.P., Shaffer S.A., Sassetti C.M. (2015). The oxidative network of *Mycobacterium tuberculosis* reveals coordination between radical detoxification systems. Cell Host Microbe.

[bib37] Pandey R., Rodriguez G.M. (2014). IdeR is required for iron homeostasis and virulence in *Mycobacterium tuberculosis*. Mol. Microbiol..

[bib38] Prodromou C., Haynes M.J., Guest J.R. (1991). The aconitase of *Escherichia coli*: purification of the enzyme and molecular cloning and map location of the gene (*acn*). J. Gen. Microbiol..

[bib39] Queval C.J., Brosch R., Simeone R. (2017). The macrophage: a disputed fortress in the battle against *Mycobacterium tuberculosis*. Front. Microbiol..

[bib40] Rodriguez G.M., Voskuil M.I., Gold B., Schoolnik G.K., Smith I. (2002). *ideR*, An essential gene in *Mycobacterium tuberculosis*: role of IdeR in iron-dependent gene expression, iron metabolism, and oxidative stress response. Infect. Immun..

[bib41] Rosser A., Stover C., Pareek M., Mukamolova G.V. (2017). Resuscitation-promoting factors are important determinants of the pathophysiology in *Mycobacterium tuberculosis* infection. Crit. Rev. Microbiol..

[bib42] Schaaf K., Hayley V., Speer A., Wolschendorf F., Niederweis M., Kutsch O., Sun J. (2016). A macrophage infection model to predict drug efficacy against *Mycobacterium tuberculosis*. Assay Drug Dev. Technol..

[bib43] Schneider C.A., Rasband W.S., Eliceiri K.W. (2012). NIH Image to ImageJ: 25 years of image analysis. Nat. Methods.

[bib44] Schwenk S., Arnvig K.B. (2018). Regulatory RNA in *Mycobacterium tuberculosis*, back to basics. Pathog. Dis..

[bib45] Serio A.W., Pechter K.B., Sonenshein A.L. (2006). *Bacillus subtilis* aconitase is required for efficient late-sporulation gene expression. J. Bacteriol..

[bib46] Smith L.J., Bochkareva A., Rolfe M.D., Hunt D.M., Kahramanoglou C., Braun Y., Rodgers A., Blockley A., Coade S., Lougheed K.E.A. (2017). Cmr is a redox-responsive regulator of DosR that contributes to *M. tuberculosis* virulence. Nucleic Acids Res..

[bib47] Soum E., Drapier J.C. (2003). Nitric oxide and peroxynitrite promote complete disruption of the [4Fe-4S] cluster of recombinant human iron regulatory protein 1. J. Biol. Inorg. Chem..

[bib48] Tang Y., Guest J.R. (1999). Direct evidence for mRNA binding and post-transcriptional regulation by *Escherichia coli* aconitases. Microbiology (Reading).

[bib49] Tong W.H., Rouault T.A. (2007). Metabolic regulation of citrate and iron by aconitases: role of iron-sulfur cluster biogenesis. Biometals.

[bib50] Turapov O., Forti F., Kadhim B., Ghisotti D., Sassine J., Straatman-Iwanowska A., Bottrill A.R., Moynihan P.J., Wallis R., Barthe P. (2018). Two faces of CwlM, an essential PknB substrate, in *Mycobacterium tuberculosis*. Cell Rep..

[bib51] Voskuil M.I., Schnappinger D., Visconti K.C., Harrell M.I., Dolganov G.M., Sherman D.R., Schoolnik G.K. (2003). Inhibition of respiration by nitric oxide induces a *Mycobacterium tuberculosis* dormancy program. J. Exp. Med..

[bib52] Walden W.E., Selezneva A.I., Dupuy J., Volbeda A., Fontecilla-Camps J.C., Theil E.C., Volz K. (2006). Structure of dual function iron regulatory protein 1 complexed with ferritin IRE-RNA. Science.

[bib53] Walden W.E., Selezneva A., Volz K. (2012). Accommodating variety in iron-responsive elements: Crystal structure of transferrin receptor 1 B IRE bound to iron regulatory protein 1. FEBS Lett..

[bib54] Warner K.D., Hajdin C.E., Weeks K.M. (2018). Principles for targeting RNA with drug-like small molecules. Nat. Rev. Drug Discov..

[bib55] Wayne L.G., Sohaskey C.D. (2001). Nonreplicating persistence of *Mycobacterium tuberculosis*. Annu. Rev. Microbiol..

[bib56] Wilson T.J., Bertrand N., Tang J.L., Feng J.X., Pan M.Q., Barber C.E., Dow J.M., Daniels M.J. (1998). The *rpfA* gene of *Xanthomonas campestris* pathovar *campestris*, which is involved in the regulation of pathogenicity factor production, encodes an aconitase. Mol. Microbiol..

[bib57] World Health Organization (2018). Global tuberculosis report. Geneva: World Health Organization. https://www.who.int/tb/publications/global_report/en/.

