# Supplementary material for: Coupling of Peptidoglycan Synthesis to Central Metabolism in Mycobacteria: Post-transcriptional Control of CwlM by Aconitase
Source: Cell Rep. 2020 Sep 29;32(13):108209. doi: 10.1016/j.celrep.2020.108209 (PMC7527780; doi:10.1016/j.celrep.2020.108209)
Supplement: Document S1. Figures S1–S5 and Tables S1 and S2 [file mmc1.pdf]

Cell Reports, Volume *32*

Supplemental Information

Coupling of Peptidoglycan Synthesis to Central Metabolism in Mycobacteria: Post-transcriptional Control of CwlM by Aconitase

Peter J. Bancroft, Obolbek Turapov, Heena Jagatia, Kristine B. Arnvig, Galina V. Mukamolova, and Jeffrey Green

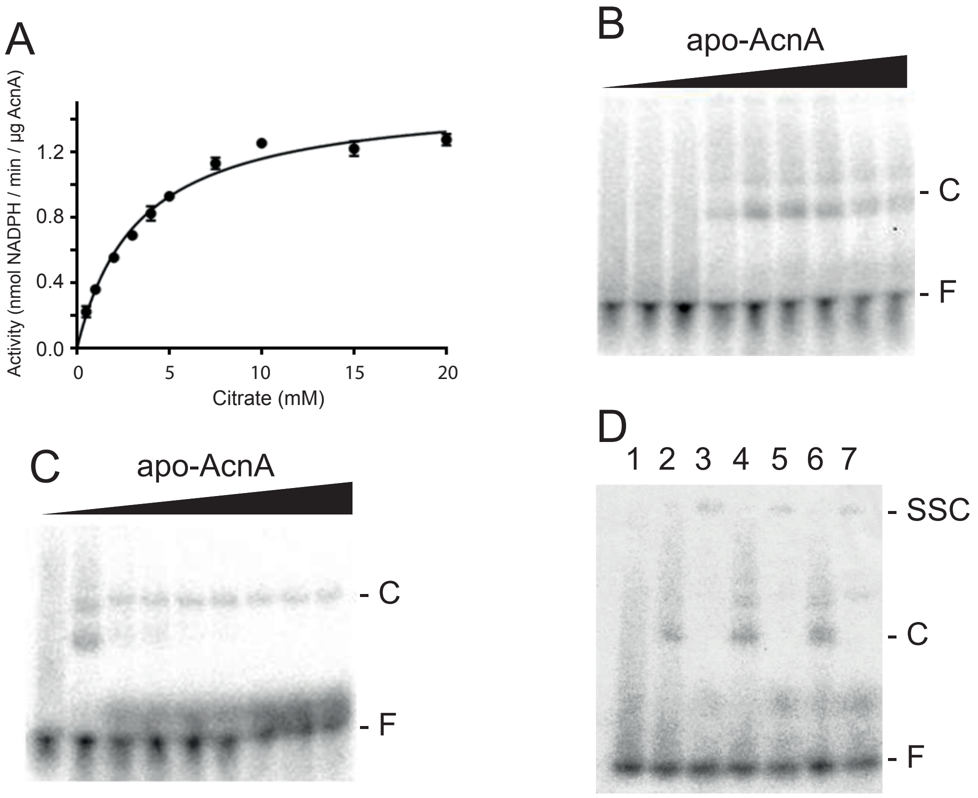

Figure S1. ***In Vitro*** **Analyses of Catalytic and RNA-binding Functions of** ***M. tuberculosis*** **Aconitase*.* Related to Figure 1.** (A) Estimation of Aconitase Kinetic Parameters. Figure displays mean and standard deviation of data obtained from assays using three enzyme preparations. The data were fitted to a Michaelis-Menten function using GraphPad Prism version 7.02. (B and C) Electrophoretic mobility shift assays showing autoradiograms of titration profile EMSAs using 10 ng wild- type IRE with (B) 0–20 µg NO-stressed holo-AcnA and (C) 0–20 µg ROS and NO-stressed holo- AcnA. The locations of the free IRE (F), the apo-AcnA:IRE complex (C) are indicated. (D) Supershift assays confirm that AcnA interacts with the *cwlM* IRE. Lanes 1-7 all contain radiolabeled *cwlM* IRE (10 ng). Lanes: 1, AcnA antiserum (Ab; 8 µg protein); 2, apo-AcnA (0.5 µM); 3, apo-AcnA (0.5 µM) plus Ab; 4, apo-AcnA (1.0 µM); 5, apo-AcnA (1.0 µM) plus Ab; 6, apo-AcnA (2.0 µM); 7, apo-AcnA (2.0 µM) plus Ab. The locations of the free IRE (F), the apo-AcnA:IRE complex (C) and the supershifted complex (SSC) are indicated next to the autoradiogram.

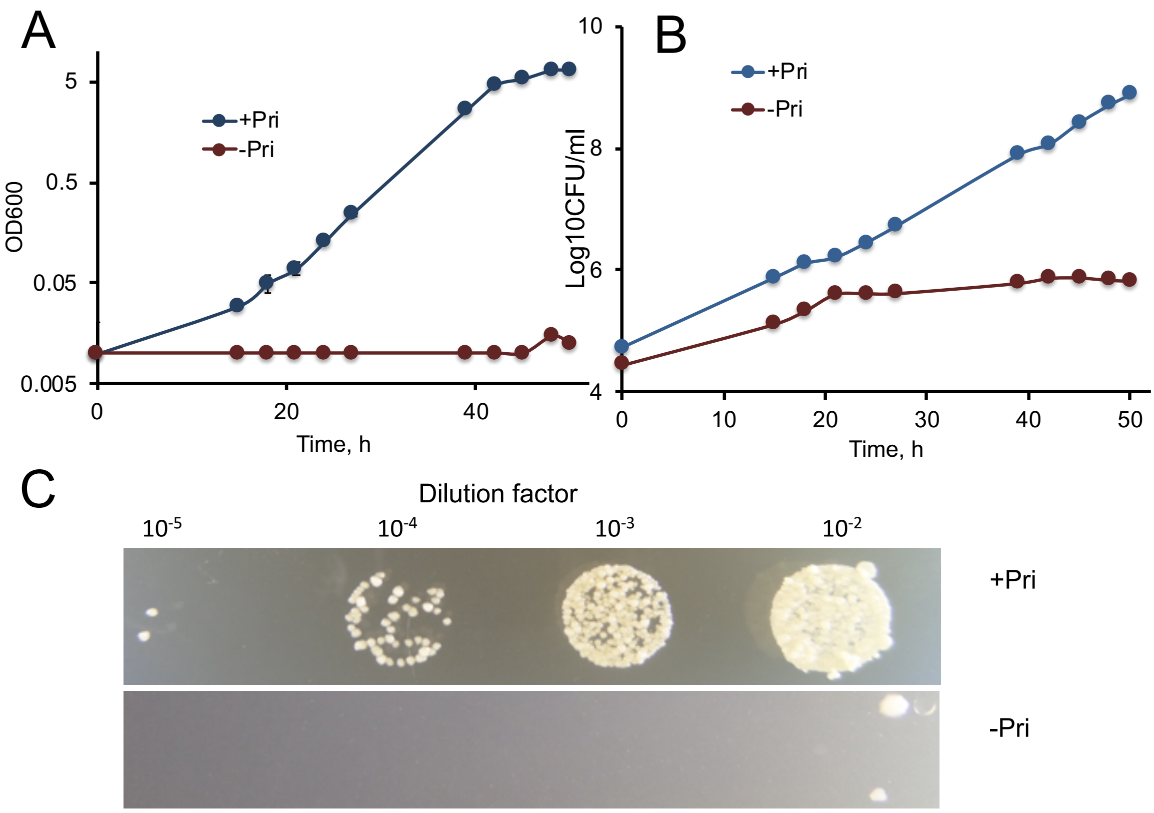

Figure S2. CwlM is Essential for Growth of *Mycobacterium smegmatis*. Related to Figure 2. (A and B) The conditional *cwlM* mutant (*Ms cwlM*-CM) was grown in supplemented 7H9 medium containing hygromycin with (+Pri, blue circles) and without (-Pri, black circles) pristinamycin at 37°C with shaking at 200 rpm. Growth was monitored by measurement of optical density at 580 nm (A) or assessment of CFU counts on 7H10 agar supplemented with hygromycin and pristinamycin (B). (C) Growth of *Ms cwlM*- CM on 7H10 agar. Representative image of colonies formed when droplets (10 µl) of serially diluted (from 10^{-2}-10^{-5}, as indicated) samples from a 39-h culture of *Ms cwlM*-CM were spotted on 7H10 agar containing hygromycin with (+Pri) or without pristinamycin (-Pri). Hygromycin and pristinamycin were added at final concentrations of 50 mg ml^{-1} and 2 mg ml^{-1}, respectively.

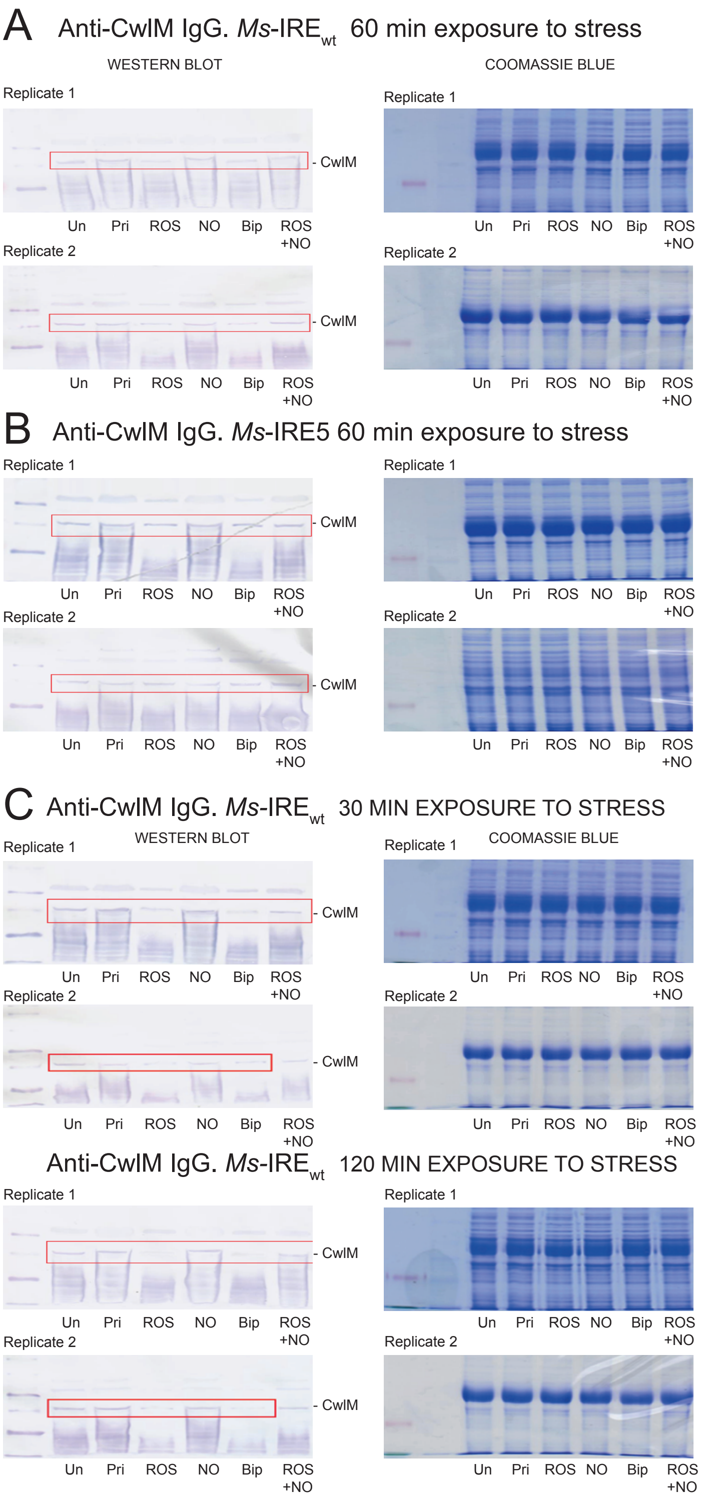

Figure S3. Western Blot Membranes and Corresponding Stained Protein Gels used for detection of CwlM protein in stressed *M. smegmatis* cultures. Related to Table 1 and Figures 4A and B. (A) Western blot and corresponding SDS-PAGE gels for cell-free extracts of *Ms*-IRE$_{wt}$ (A) and *Ms*-IRE5 (B) after cultures were exposed to the indicated stresses for 60 min or *Ms*-IRE$_{wt}$ cultures exposed to the indicated stresses for 30 and 120 min. (A-C) Oxidative stress (ROS); nitrosative stress (NO); iron-starvation (Bipyridyl); oxidative and nitrosative stress (ROS+NO). Untreated cultures and cultures in which the chromosomal *M. smegmatis cwlM* was induced by addition of pristinamycin (Pri) are also shown. The blots were developed with a polyclonal anti-CwlM antibody and hence all forms of CwlM are detected. The red rectangles indicate the sections of the western blots that were analysed for determination of protein abundance shown in Table 1 and Figure 4A and B.

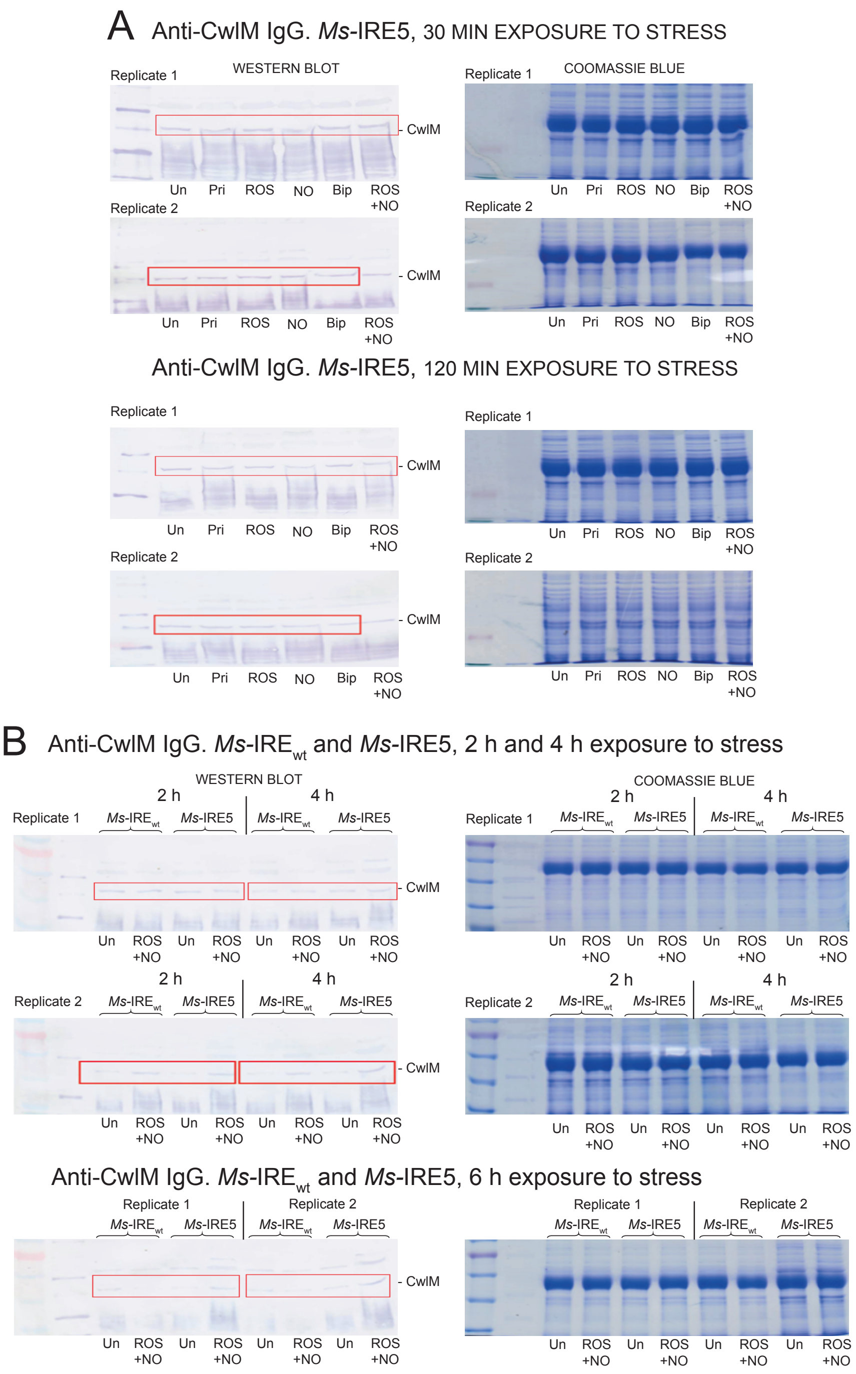

Figure S4. Western Blot Membranes and Corresponding Stained Protein Gels used for detection of CwlM protein in stressed *M. smegmatis* cultures. Related to Figures 4A, B and C. (A) Western blot and corresponding SDS-PAGE gels for cell-free extracts of *Ms*-IRE5 after cultures were exposed to the indicated stresses for 30 and 120 min. Oxidative stress (ROS); nitrosative stress (NO); iron-starvation (Bipyridyl); oxidative and nitrosative stress (ROS+NO). Untreated cultures and cultures in which the chromosomal *M. smegmatis cwlM* was induced by addition of pristinamycin (Pri) are also shown. (B) Western blot and corresponding SDS-PAGE gels for cell-free extracts of *Ms*-IRE$_{wt}$ and *Ms*-IRE5 after cultures were exposed to a combination of oxidative and nitrosative stress (ROS+NO) or were left untreated (Un) for 2, 4 and 6 h. (A-C) The blots were developed using a polyclonal anti-CwlM antibody and hence all forms CwlM were detected. The red rectangles indicate the sections of the western blots that were analysed for determination of protein abundance shown in Figures 4A, B and C.

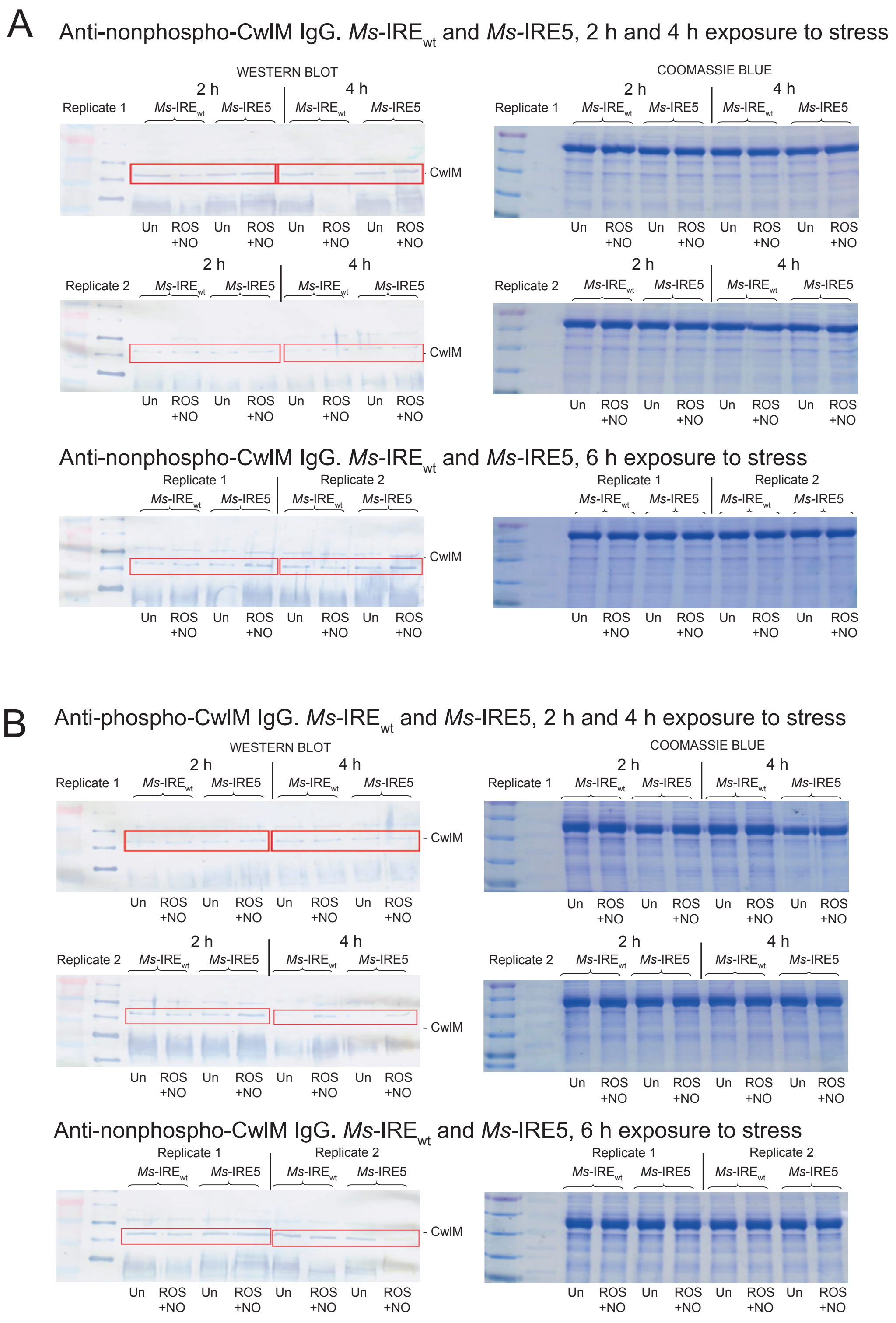

Figure S5. Western Blot Membranes and Corresponding Stained Protein Gels used for detection of CwlM protein in stressed *M. smegmatis* cultures. Related to Figure 4 C. (A) Western blot and corresponding SDS-PAGE gels for cell-free extracts of *Ms*-IRE$_{wt}$ and *Ms*-IRE5 after cultures were exposed to a combination of oxidative and nitrosative stress (ROS+NO) or were left untreated (Un) for 2, 4 and 6 h. (A) The blots were developed using antibodies specific for non-phosphorylated CwlM. (B) The blots were developed using antibodies specific for phospho-CwlM. The red rectangles indicate the sections of the western blots that were analysed for determination of CwlM abundance shown in Figure 4 C.

Table S1. Relative Abundances of CwlM in *Ms*-IRE$_{WT}$ and *Ms*-IRE5 Cultures. Related to Table 1 and Figure 4.

Condition	Replicate 1	Replicate 2	Mean	95% CI
**30 min WT Figure 4A**				
Pristinamycin	0.988	2.098	1.543	0.796
ROS	0.175	0.479	0.327	0.211
NO	1.433	0.905	1.169	0.366
BIP	0.473	0.231	0.352	0.167
**30 min IRE5 Figure 4A**				
Pristinamycin	1.764	1.124	1.444	0.440
ROS	1.332	0.868	1.100	0.322
NO	0.920	1.718	1.319	0.533
BIP	1.407	0.725	1.066	0.473
**60 min WT Table 1**				
Pristinamycin	2.092	1.075	1.583	0.705
ROS	0.726	0.478	0.602	0.172
NO	1.800	0.711	1.254	0.753
BIP	0.784	0.358	0.571	0.295
ROS+NO	1.633	1.047	1.340	0.405
**60 min IRE5 Table 1**				
Pristinamycin	1.606	2.240	1.923	0.44
ROS	0.694	1.409	1.051	0.495
NO	1.546	0.670	1.108	0.607
BIP	1.357	0.870	1.114	0.338
ROS+NO	1.022	2.225	1.624	0.834
**120 min WT Figure 4B**				
Pristinamycin	1.461	1.303	1.382	0.110
ROS	0.366	0.176	0.271	0.132
NO	1.270	1.312	1.291	0.030
BIP	0.484	0.108	0.296	0.260
**120 min IRE5 Figure 4B**				
Pristinamycin	0.988	0.844	0.916	0.099
ROS	0.820	0.803	0.82	0.012
NO	1.072	0.664	0.870	0.283
BIP	0.635	1.181	0.908	0.379
**2 h WT NO/ROS Figure 3C**				
Anti-CwlM	0.860	1.289	1.074	0.297
Anti-CwlM~P	0.637	0.654	0.645	0.012
Anti-nonphospho-CwlM	0.670	1.188	0.929	0.359
**2 h IRE5 NO/ROS Figure 4C**				
Anti-CwlM	1.145	1.260	1.202	0.08
Anti-CwlM~P	1.114	1.453	1.283	0.235
Anti-nonphospho-CwlM	0.894	1.299	1.097	0.281
**4 h WT NO/ROS Figure 3C**				
Anti-CwlM	0.569	1.301	0.935	0.507
Anti-CwlM~P	0.683	0.968	0.825	0.198
Anti-nonphospho-CwlM	0.399	0.499	0.449	0.069
**4 h IRE5 NO/ROS Figure 4C**				
Anti-CwlM	1.355	1.750	1.553	0.274
Anti-CwlM~P	0.834	1.052	0.943	0.151
Anti-nonphospho-CwlM	1.727	1.139	1.433	0.408
**6 h WT NO/ROS Figure 4C**				
Anti-CwlM	0.359	0.634	0.497	0.19
Anti-CwlM~P	0.899	0.646	0.773	0.176
Anti-nonphospho-CwlM	0.463	0.624	0.543	0.112
**6 h IRE5 NO/ROS Figure 4C**				
Anti-CwlM	1.800	2.366	2.083	0.392

Anti-CwlM~P	1.530	0.670	1.100	0.596
Anti-nonphospho-CwlM	2.920	2.075	2.497	0.585

*Cultures were incubated under the indicated conditions before preparation of cell lysates for western blot analysis. Fold difference was calculated relative to the corresponding untreated cultures as described in *STAR Methods*. Mean values ± 95% confidence intervals (n=2) are shown.

Table S2. Oligonucleotides used in this study. Related to Figures 1-4 and S1-S2.

Name	Sequence 5'-3'	Comments
For SDM		
PB01	CTGCGCTGTGGGGACCGGAGTGCGGCCGTCACCG	This paper
PB02	CGGTGACGGCCGCACTCCGGTCCCCACAGCGCAG	This paper
PB03	GGGGACCGGAGTGCAGCAGTGACCGAGATCCGGG	This paper
PB04	CCCGGATCTCGGTCACTGCTGCACTCCGGTCCCC	This paper
For annealing and ligating into pGEM-3Zf for IVT		
PB05	AATTCGCTGTGGCGACCGCAGTGCGGCCGTCACCGAGG	This paper
PB06	AATTCCTCGGTGACGGCCGCACTGCGGTCGCCACAGCG	This paper
PB07	AATTCGCTGTGGGGACCGGAGTGCGGCCGTCACCGAGG	This paper
PB08	AATTCCTCGGTGACGGCCGCACTCCGGTCCCCACAGCG	This paper
PB09	AATTCGCTGTGGGGACCGGAGTGCAGCAGTGACCGAGG	This paper
PB10	AATTCCTCGGTCACTGCTGCACTCCGGTCCCCACAGCG-	This paper
For RT-qPCR amplification of *rrsA*		
QRRSF	AACTGACGCTGAGGAGCGAAAG	This paper
QRRSR	TCCCAAGGAAGGAAACCCACAC	This paper
For RT-qPCR amplification of *cwlM*		
QCWLF	TTTCACTTCGGCAACTCGCACG	This paper
QCWLR	TTCGACCATGCACACGGCAATC	This paper
For sequencing pMV306		
PMV306F	TGGTATCTTTATAGTCCTGTC-	This paper
PMV306R2	TAGTTAACTACGTCGACATCGA	This paper
For sequencing pET28a.Acn		
PB00	AAGCTGTGACTAGCAAATCTGTGAACTC	This paper
LS62	CTCGAGTCAGCCTGACTTCAGTATG	This paper
LS63	GTCCTGCACCAACACCTCCAACCCC	This paper
LS64	CAATCGTGGTCTTCACCCACGGC	This paper
LS65	CCCGCGAAATTAATACGACTCAC	This paper
LS66	CGGGCTTTGTTAGCAGCCGGATC	This paper
For sequencing pGEM-3Zf		
M13F	GTTTTCCCAGTCACGAC	This paper
M13R	CAGGAAACAGCTATGAC	This paper
For generation of pAZI9479::*cwlM*$_{Ms}$		
MSMEG6935F	ACTCCATGGAGGGGCCCCGTATGTCGAGTCTGCGT	This paper
MSMEG6935R	ATAGGATGCATCGGAGGGGCTGCGGTTCGGCGGGC	This paper
For generation pAZI9479::*cwlM*$_{Mt}$		
CMRv3915F	ACTGCCATGGGCCCGAGTCCGCGCGAA	Turapov et al., 2018
CMRv3915R	ACGTGCATGCTCATGCGTCGGACGGACTACG	Turapov et al., 2018
For generation of pMV306::*cwlM*$_{Mt}$ (WT)		
3915pMV306F2	ACTGGTACC AGCCGGTGAAACGAATCGTT	Turapov et al., 2018
3915pMV306R	TACAAGCTTTAAGAACCGCCGAGTCTACC	Turapov et al., 2018
